# Exchangeable coalescents beyond the Cannings class

**DOI:** 10.1007/s00285-024-02173-x

**Published:** 2024-12-17

**Authors:** Arno Siri-Jégousse, Alejandro H. Wences

**Affiliations:** 1 IIMAS, Universidad Nacional Autónoma de México, Mexico City, Mexico; 2 LAAS - CNRS, Université de Toulouse, Toulouse, France

**Keywords:** Coalescent processes, Neutral evolution models, Non-heritability, Exponential models, 60F05, 60G09, 60J05, 60J90, 92D15

## Abstract

We propose a general framework for the study of the genealogy of neutral discrete-time populations. We remove the standard assumption of exchangeability of offspring distributions appearing in Cannings models, and replace it by a less restrictive condition of non-heritability of reproductive success. We provide a general criterion for the weak convergence of their genealogies to $\Xi$-coalescents, and apply it to a simple parametrization of our scenario (which, under mild conditions, we also prove to essentially include the general case). We provide examples for such populations, including models with highly-asymmetric offspring distributions and populations undergoing random but recurrent bottlenecks. Finally we study the limit genealogy of a new exponential model which, as previously shown for related models and in spite of its built-in (fitness) inheritance mechanism, can be brought into our setting.

## Introduction

Coalescent processes appear as the natural limits that describe the genealogy of evolving population models. In fact, much of the research on population models focuses on establishing this connection, often as weak limits under appropriate time scales and/or time changes. A primordial class of population models are Cannings models (Cannings 1974, 1975). These are able to accommodate most biological premises that fall under the assumption of neutral evolution. The key assumption of these models is that the offspring distribution of the parents in any generation should be exchangeable (invariant under permutations); in particular all the parents have the same reproductive success, or fitness, in distribution. Due to their wide applicability and manageability, Cannings models are among the most studied population evolution models. The genealogy of Cannings models was described in whole generality, and in terms of coalescent processes, in the foundational work of Möhle and Sagitov (2001). After this, a wide literature was developed on the study of limit genealogies of evolution models. Some are direct applications e.g. Huillet and Möhle (2021), or deal with a priori more complex models that can be reduced to Cannings models (Schweinsberg 2003; Cortines and Mallein 2017). Others are modifications, such as models with varying population sizes (Möhle 2002; Kaj and Krone 2003; Freund 2020) or diploid reproduction (Möhle and Sagitov 2003; Birkner et al. 2018), using heuristics close to those of Möhle and Sagitov (2001). Finally some works use different techniques such as duality, e.g. in the case of populations suffering recurrent bottlenecks (González Casanova et al. 2022) or overlapping generations (Casanova et al. preprint arXiv:2210.05819).

Here we study a generalization of Cannings models, the so-called Asymmetric Cannings (AC) models, that, nonetheless, still fall under the umbrella of neutral evolution. Our results provide weaker conditions for the main result in Möhle and Sagitov (2001), expanding its applicability and providing a better insight into neutral evolution from a mathematical perspective. Having an accurate understanding of the neutral case is paramount for the theory and applications where the effect of natural selection is studied. One of our main contributions is the change in focus in neutral models from the exchangeability (symmetry) condition on offspring distributions, to the weaker condition of non-heritability of reproductive success. It is in fact from the latter property, and not the former, from which neutrality stems; both, from biological (heritability is a necessary condition for natural selection to occur, whereas asymmetric reproductive success is not sufficient) and mathematical perspectives.

Indeed, as argued in Möhle and Sagitov (2001), the assumed exchangeability of the offspring distribution in Cannings models ensures that the partition-valued ancestry process evolves, in distribution, through sequential “putting balls into boxes” steps. In fact it is from this last property from which the Markovicity and exchangeability of the ancestry process stem. In this work we thus remove the exchangeability condition on the offspring distribution of Cannings models, and instead model neutral evolution by removing any form of inheritance, letting children choose parents in an exchangeable way. In other words, we allow individuals in any given generation to have different offspring distributions, but impose that these differences in reproductive success stem from non-heritable traits. An example of this would be a population whose individuals randomly explore different ecological niches in every new generation, resulting in non-heritable asymmetries in reproductive success. Formally, AC models consist of a population with constant size *N* evolving in discrete time. Letting ${\varvec{\xi }}^{(N)}(t){:=}\left( \xi _{1}^{(N)}(t),\cdots ,\xi _{N}^{(N)}(t)\right)$ be the offspring sizes of each parent in generation $t\in \mathbb {N}$, the framework makes the following assumptions:

1. **Static environment.** The sequence of vectors of offspring sizes $\big ({\varvec{\xi }}^{(N)}(t)\big )_{t\ge 0}$ is i.i.d..
2. **Non-heritability.** Children choose parents in an exchangeable way; i.e. the random vector of indices indicating the parent of each child in the previous generation must be exchangeable (and the number of repetitions of each index should of course comply with ${\varvec{\xi }}^{(N)}$).

As a special case, we focus on a parametrization of this scenario, the class of Asymmetric Wright–Fisher (AWF) models, in which the offspring distribution is assumed to be multinomial. The key difference between multinomial and general offspring distributions (within our framework), is that in AWF models children choose parents via a sampling with replacement, whereas in the general AC case the sampling is done without replacement. Taking this distinction into account one can easily recast our results from AWF models to AC models. In the AWF framework, children of each generation choose their parent according to independent copies of a random probability vector ${\varvec{\eta }}^{(N)}=(\eta ^{(N)}_1,\dots ,\eta ^{(N)}_N)$, and the distribution of the offspring number of individuals at a given generation is multinomial with parameters $(N,{\varvec{\eta }}^{(N)})$.

In this work we provide a general criterion for the weak convergence of discrete-time coalescent processes to $\Xi$-coalescents as $N\rightarrow \infty$; which in particular applies to the genealogy of AC and AWF populations. This in fact follows by a careful study of the corresponding arguments in Möhle and Sagitov (2001) for Cannings populations. In the case of AC models, one may argue that any vector of offspring sizes $(\xi _{1}^{(N)},\dots ,\xi _{N}^{(N)})$ becomes exchangeable once we randomly permute its entries, thus effectively rendering any model with non-heritability, and in fact more general models, into a Cannings model. In other words, the distribution of the genealogy of the AC model is equal to that of the Cannings model with offspring sizes $(\xi _{\sigma (1)}^{(N)},\dots ,\xi _{\sigma (N)}^{(N)})$ where $\sigma$ is a uniform permutation of [*N*]. This loss of information on the asymmetric reproductive success of the population will, however, over-complicate the study of its genealogy. We are able to avoid this unnecessary step and harness the asymmetry of the reproduction laws to more easily derive the limit genealogy of the population.

We also show that, in most cases, the difference between sampling with and without replacement becomes negligible, making the dynamics of AC and AWF models essentially equivalent. This is a general result in the same spirit as Huillet and Möhle (2021), where the authors study the sampling with replacement version of the model in Schweinsberg (2003), see also González Casanova et al. (2023) for a multinomial version of Cannings models with seed bank effects.

The new class of asymmetric models and the techniques developed in this paper have some straightforward applications to population models that do not satisfy Cannings assumption of exchangeability. A first example is given by the celebrated model developed in Eldon and Wakeley (2006) for populations with skewed offspring distributions where only one individual per generation reproduces. Another example of asymmetric reproduction is given by (a slight modification of) the model in González Casanova et al. (2022) for populations suffering recurrent bottlenecks, for which a whole generation can be produced by a small amount of individuals from the previous one.

A more interesting consequence of our method is its applicability to a certain class of non-neutral models. Exponential models (Brunet and Derrida 1997) provide a surprising example where mutation and selection are incorporated and, however, the built-in inheritance mechanism in fact disappears due to mathematical transformations (mainly Poisson point processes identities). These models can therefore be studied under the AWF framework. The exponential model we are considering here is an extension that combines those of Cortines and Mallein (2018, 2017). As for most models incorporating strong selective pressure, their limit genealogy is described by the coalescent first introduced by Bolthausen and Sznitman (1998). The Bolthausen–Sznitman coalescent has been proposed as a null model for the genealogies of rapidly adapting populations (see e.g. Neher and Hallatschek (2013)). Many examples in the literature have established this link, such as those in Neher and Hallatschek (2013), Schweinsberg (2017), Cortines and Mallein (2018), Cortines and Mallein (2017), Berestycki et al. (2013), Schertzer and Wences (2023), to mention a few. To the best of our knowledge, our model is the first example in which the convergence to the Bolthausen–Sznitman coalescent holds in a time scale other than logarithmic on the size *N* of the population.

This paper is structured as follows. In Sect. 2, we establish the main convergence results for the genealogies of both AC and AWF models, but also establish a correspondence so that more tractable results for AWF models can be used in the AC case. In Sect. 3, we illustrate how we can exploit the asymmetry when the limit genealogy is a $\Lambda$-coalescent, simplifying the proofs and generalizing similar results found in the literature. The same techniques can also be used for the general $\Xi$-coalescent case; for simplicity and space constraints we do not include such development here. Section 4 is devoted to the application of our main results to neutral asymmetric models of skewed offspring and bottlenecks. In Sect. 5 we focus on a particular example of an AWF model where the family frequencies are obtained from a Poisson–Dirichlet partition, and establish a relation with Beta-coalescents in this case. Thanks to this new result, we will be able to study a new class of exponential models.

## The genealogy of models with non-heritability

### Weak convergence of coalescent processes

In this section we present our main weak convergence theorem for general discrete-time coalescent processes. It is in fact a re-framing of the main theorem in Möhle and Sagitov (2001) which, nonetheless, uncovers its generality and its possible application to population models beyond the Cannings class.

Let us first introduce some notation and recall some terminology from Bertoin (2006). Let $\mathscr {P}_{n}$ (resp. $\mathscr {P}_{\infty }$) be the set of partitions of $[n]{:=}\{1,\cdots ,n\}$ (resp. $\mathbb {N}$) endowed with their usual topology (Lemma 2.6 in Bertoin (2006)), and $\textbf{0}_n\in \mathscr {P}_{n}$ (resp. $\textbf{0}_\infty$) be the partition of singletons. For any partition $\pi$, denote its blocks (its composing elements), ordered by their least elements, by $\pi _1,\pi _2,\cdots$. More precisely,

- $$\pi _i\in \pi $$ for all $$i\in \mathbb {N}$$
- $$\pi =\{ \pi _i :1\le i \le \infty \}$$
- $$\min \{k: k\in \pi _j\} \ge \min \{k: k\in \pi _i\}$$ for all $$j\ge i $$.

Denote the cardinality of any set *A* by $\left|A \right|$. Write ${{\,\textrm{Coag}\,}}(\cdot ,\cdot )$ for the usual coagulation operator on $\mathscr {P}_{n}\times \mathscr {P}_{n}$ (Definition 4.2 in Bertoin (2006)): if $\pi '\in \mathscr {P}_{m}$ and $\pi \in \mathscr {P}_{n}$ are such that $\left|\pi \right| \le m\le \infty$ we say that the pair $(\pi ,\pi ')$ is admissible, and define the coagulation of $\pi$ by $\pi '$ as

$$\begin{aligned} {{\,\textrm{Coag}\,}}(\pi ,\pi ')=(\hat{\pi }_1,\hat{\pi }_2,\cdots ), \end{aligned}$$

where

$$\begin{aligned} \hat{\pi }_k {:=}\bigcup _{j\in \pi '_k} \pi _j. \end{aligned}$$

Of course $\pi _j$ is set to $\emptyset$ if $j>\left|\pi \right|$.

Consider a discrete-time coalescent process $\left( \varPi ^{(N,n)}_t\right) _{t\in \mathbb {N}}$ constructed as

1 $$\begin{aligned} \varPi ^{(N,n)}_t{:=}{\left\{ \begin{array}{ll} \textbf{0}_n, & t=0,\\ {{\,\textrm{Coag}\,}}\left( \varPi ^{(N,n)}_{t-1},\tilde{\Pi }^{(N)}_t\right) , & t > 0, \end{array}\right. } \end{aligned}$$

where the family of coagulation increments $\left( \tilde{\Pi }^{(N)}_h\right) _{h\in \mathbb {N}}$ is i.i.d. and each partition $\tilde{\Pi }^{(N)}_h$ is exchangeable (i.e., its distribution is invariant under any random permutation of the elements in its blocks, see e.g. section 2.3.2 Bertoin (2006)). We are interested in the weak limit as $N\rightarrow \infty$ of $\big (\varPi ^{(N,n)}_{\lfloor t/c_N\rfloor }\big )_{t\ge 0}$. The correct time scale, as is for Cannings models, is given by the probability $c_N$ that two randomly chosen individuals have the same parent in the previous generation. Thanks to exchangeability, this probability can be written in terms of the indices 1 and 2 as

2 $$\begin{aligned} c_N= \mathbbm {P}\left( 1 \text { and }2\text { belong to the same block of }\tilde{\Pi }^{(N)}_h\right) . \end{aligned}$$

We now introduce some additional notation. Let $\mathscr {P}_{[0,1]}$ be the space of mass partitions

$$\begin{aligned} \mathscr {P}_{[0,1]}{:=}\{(\rho _1,\rho _2,\cdots ):\rho _1\ge \rho _2\ge \cdots , \text { and }\sum _{i=1}^\infty \rho _i \le 1\} \end{aligned}$$

endowed with the $\textsf {L}^{\infty }$ topology, and consider a $\Xi$-coagulation measure on $\mathscr {P}_{[0,1]}$ (see Section 4.2.4 in Bertoin (2006)). Let ${\varvec{Q}}^{(n)}_{\Xi }$ be the coagulation matrix of the $\Xi$-coalescent with values on $\mathscr {P}_{n}$; if $(\pi ,\pi ')$ is an admissible pair of partitions, ${\varvec{Q}}^{(n)}_{\Xi }(\pi ,{{\,\textrm{Coag}\,}}(\pi ,\pi '))$ gives the rate at which a $\Xi$-coalescent jumps from $\pi$ to ${{\,\textrm{Coag}\,}}(\pi ,\pi ')$. If $I\equiv I_{\pi '}$ is the set of indices *i* such that $\left|\pi '_i \right|=1$, and for ${\varvec{\rho }}\in \mathscr {P}_{[0,1]}$ we write $\rho _0=1-\sum _{i=1}^\infty \rho _i$, then these rates are given by

3 $$\begin{aligned} {\varvec{Q}}^{(n)}_{\Xi }(\pi ,{{\,\textrm{Coag}\,}}(\pi ,\pi ')) = \int _{\mathscr {P}_{[0,1]}} \sum _{\begin{array}{c} i_1,\cdots ,i_{\left|\pi ' \right|}> 0\\ \text {all distinct} \end{array}} \prod _{j\in I} (\rho _{i_j}+\rho _0) \prod _{j\not \in I}\rho _{i_j}^{\left|\pi '_{j} \right|} \frac{\Xi (d{\varvec{\rho }})}{\sum _{i=1}^\infty \rho _i^2}. \end{aligned}$$

(see also e.g. Equation (11) in Schweinsberg (2000)). The above expression is a consequence of the well-known paint-box construction (see e.g. section 2.3.2 in Bertoin (2006)) of random exchangeable partitions of [*N*] from a mass partition ${\varvec{\rho }}$: one partitions the unit interval into consecutive non-overlapping sub-intervals of sizes $\rho _1,\rho _2,\cdots ,$ respectively and, letting $(U_i)_{1\le i\le N}$ be i.i.d. standard uniform random variables, one constructs a partition of [*N*] by putting together in the same block all those indices *i* such that $U_i$ falls on the same interval of type $[\sum _{j=1}^k \rho _j, \sum _{j=1}^{k+1} \rho _j)$; whereas all those indices whose $U_i$ falls on the interval $[1-\rho _0,1)$ become singleton blocks. In equation (3), it is the coagulation increment $\pi '$ that is paint-box constructed from a mass partition ${\varvec{\rho }}$ that is chosen at rate $\frac{\Xi (d{\varvec{\rho }})}{\sum _{i=1}^\infty \rho _i^2}$. Note that, the random variables $U_i$ being independent, and conditional on the value of ${\varvec{\rho }}$, the sum of products appearing in (3) simply computes the probability that the paint-box construction directed by ${\varvec{\rho }}$ results in the partition $\pi '$. Finally, for any coagulation matrix ${\varvec{Q}}^{(n)}$ operating on $\mathscr {P}_{n}$, we use the abbreviation ${\varvec{Q}}^{(n)}(\tilde{\pi }){:=}{\varvec{Q}}^{(n)}(\textbf{0}_n,\tilde{\pi })$.

The following result was previously stated and proved in Möhle and Sagitov (2001) for the genealogies of Cannings models. Here we emphasize that the same statement, and in fact the same proofs, remain valid (verbatim) in the context of the more general discrete-time coalescent processes defined as in (1). We include the corresponding argument for completion.

#### Theorem 2.1

Fix $$n\in \mathbb {N}$$. Let

$$\begin{aligned} {\varvec{P}}^{(N,n)}({\pi , {{\,\textrm{Coag}\,}}(\pi ,\tilde{\pi })})\equiv {\varvec{P}}^{(N,n)}(\tilde{\pi }) = \mathbbm {P}(\tilde{\Pi }^{(N)}_h=\tilde{\pi }) \end{aligned}$$

be the transition matrix of a discrete-time coalescent as defined in (1). Also, let ${\varvec{Q}}^{(n)}_{\Xi }$ be a coagulation matrix associated to a measure $\Xi$ on $\mathscr {P}_{[0,1]}$. Assume that

4 $$\begin{aligned} {\varvec{P}}^{(N,n)}(\tilde{\pi }) = {\left\{ \begin{array}{ll} c_N{\varvec{Q}}^{(n)}_{\Xi }(\tilde{\pi }) + o\big (c_N\big ) & \text { if }\tilde{\pi }\in \mathscr {P}_{n}\setminus \{\textbf{0}_n\}\\ 1-c_N\sum _{\pi \in \mathscr {P}_{n}\setminus \textbf{0}_n} {\varvec{Q}}^{(n)}_{\Xi }(\pi ) + o\big (c_N\big )& \text { if }\tilde{\pi }=\textbf{0}_n. \end{array}\right. } \end{aligned}$$

(i) If $c_N\rightarrow c>0$ as $N\rightarrow \infty$, then $\left( \varPi ^{(N,n)}_{t}\right) _{t\in \mathbb {N}}$ converges weakly in the product topology for $(\mathscr {P}_{n})^\mathbb {N}$ to a Markov chain with initial state $\textbf{0}_n$ and transition matrix ${\varvec{I}}+ c{\varvec{Q}}^{(n)}_{\Xi }$.
(ii) If $c_N\rightarrow 0$ as $N\rightarrow \infty$, then $\big (\varPi ^{(N,n)}_{\lfloor t/c_N\rfloor }\big )_{t\ge 0}$ converges weakly in the Skorohod space $D([0,\infty ), \mathscr {P}_{n})$ to the $\Xi$-coalescent with initial state $\textbf{0}_n$.

#### Proof

To prove (i) note that (4) plus the condition $c_N\rightarrow c>0$ imply $\lim _{N\rightarrow \infty }{\varvec{P}}^{(N,n)} = {\varvec{I}}+ c{\varvec{Q}}^{(n)}_{\Xi }$ which in turn gives convergence of the finite dimensional distributions. The latter is equivalent to weak convergence on $\mathscr {P}_{n}^\mathbb {N}$ (see Example 2.6 in Billingsley (1999)).

Let us now prove (ii). Given that both $\big (\varPi ^{(N,n)}_{\left[ t/c_N\right] }\big )_{t\ge 0}$ and the $\Xi$-coalescent are Feller processes, we need only prove convergence of their semigroups (Theorem 2.5 in Ethier and Kurtz (1986)). By means of the equality

$$\begin{aligned} \left( {\varvec{P}}^{(N,n)}\right) ^{k} -&\left( {\varvec{I}}+ c_N{\varvec{Q}}^{(n)}_{\Xi }\right) ^{k}\\&= \sum _{i=1}^k \left( {\varvec{P}}^{(N,n)}\right) ^{i-1}({\varvec{P}}^{(N,n)} - \left( {\varvec{I}}+ c_n{\varvec{Q}}^{(n)}_{\Xi }\right) )\left( {\varvec{I}}+ c_N{\varvec{Q}}^{(n)}_{\Xi }\right) ^{k-i} \end{aligned}$$

(which follows by telescoping the sum on the right hand side), we compute, for every $t>0$,

$$\begin{aligned}&\left\Vert \left( {\varvec{P}}^{(N,n)}\right) ^{\left[ \frac{t}{c_N}\right] } - ({\varvec{I}}+ c_N{\varvec{Q}}^{(n)}_{\Xi })^{\left[ \frac{t}{c_N}\right] } \right\Vert \\&\quad \le \left[ \frac{t}{c_N}\right] \left\Vert {\varvec{P}}^{(N,n)} - ({\varvec{I}}+ c_N{\varvec{Q}}^{(n)}_{\Xi }) \right\Vert (1 + \left\Vert \textbf{P}- ({\varvec{I}}+ c_N{\varvec{Q}}^{(n)}_{\Xi }) \right\Vert )^{\left[ \frac{t}{c_N}\right] }\\&\quad \le \left[ \frac{t}{c_N}\right] \left\Vert {\varvec{P}}^{(N,n)} - ({\varvec{I}}+ c_N{\varvec{Q}}^{(n)}_{\Xi }) \right\Vert \exp \left\{ \left[ \frac{t}{c_N}\right] \left( o\big (c_N\big ) + o\big (c_N^2\big ) \right) \right\} \\&\quad \overset{N\rightarrow \infty }{\longrightarrow }\ 0; \end{aligned}$$

where $\left\Vert \cdot \right\Vert$ refers to the usual operator norm, and we have used (4). Therefore

$$\begin{aligned} \lim _{N\rightarrow \infty } \left( {\varvec{P}}^{(N,n)}\right) ^{\left[ \frac{t}{c_N}\right] } = \lim _{N\rightarrow \infty } \left( {\varvec{I}}+ c_N{\varvec{Q}}^{(n)}_{\Xi }\right) ^{\left[ \frac{t}{c_N}\right] } = e^{t{\varvec{Q}}^{(n)}_{\Xi }}. \end{aligned}$$

$$\square $$

### The Asymmetric Cannings framework

Consider a random population satisfying the static environment and non-heritability conditions of the AC model described above. To construct its genealogy, set a time horizon $T>0$ and for $0\le t \le T$ let $A^{(N)}_t$ be the random partition of [*N*] resulting from grouping all the individuals at time *T* according to their common ancestors at time $T-t$. Similarly, for $n\le N$, let $(A^{(N,n)})_{0\le t\le T}$ be the genealogy of a random sample of *n* individuals from the population at time *T* constructed in the same way. The static environment and non-heritability conditions in AC models allow us to study the genealogy $(A^{(N,n)}_t)_{0\le t\le T}$ through a coalescent process constructed as in (1). Indeed, the distribution of the coagulation increments $\tilde{\Pi }^{(N)}_h$ should simply be set to the distribution of the random partition in the AC model resulting from grouping the first *n* individuals (or, by exchangeability, any random sample of *n* individuals) in generation $T-h+1$ according to their parents in the previous generation $T-h$. Using the non-heritability condition one easily sees that the coagulation increments $\tilde{\Pi }^{(N)}_h$ do indeed coincide in distribution with those of $(A^{(N,n)}_t)_{0\le t\le T}$. On the other hand, the static environment condition ensures that the sequence $\tilde{\Pi }^{(N)}_h$ is i.i.d., and thus also the Markovicity of the process $(A^{(N,n)}_t)_{0\le t\le T}$. Putting both observations together we see that $(\varPi ^{(N,n)}_{t})_{0\le t\le T}$ and $(A^{(N,n)}_t)_{0\le t\le T}$ coincide in distribution. Furthermore, this allows us to take $T=\infty$ in $(\varPi ^{(N,n)}_{t})_{0\le t\le T}$ to describe (in distribution) the genealogy of the AC model as the time horizon grows to infinity.

In the AC framework the matrix of transition probabilities ${\varvec{P}}^{(N,n)}$ can be expressed as

$$\begin{aligned} {\varvec{P}}^{(N,n)}(\tilde{\pi }) = \mathbbm {E}\left[ \sum _{\begin{array}{c} i_1,\dots ,i_{\left|\tilde{\pi } \right|} \\ \text {all distinct} \end{array}} \frac{\left( \xi _{i_1}^{(N)}\right) _{b_1} \cdots \left( \xi _{i_{\left|\tilde{\pi } \right|}}^{(N)}\right) _{b_{\left|\tilde{\pi } \right|}}}{(N)_{n}} \right] \end{aligned}$$

where for $a\in \mathbb {R}$ and $b\in \mathbb {N}$ we have written $(a)_b= a(a-1)\cdots (a-b+1)$, and $b_k=\left|\tilde{\pi }_k \right|$. In particular, the probability $c_N$ that two randomly chosen individuals share the same parent in the previous generation is given by

5 $$\begin{aligned} c_N= \frac{1}{N(N-1)}\sum _{i=1}^N \mathbbm {E}\left[ \xi _{i}^{(N)}\left( \xi _{i}^{(N)}-1\right) \right] . \end{aligned}$$

Plugging these into (4), Theorem 2.1 yields a general criterion for the convergence of the genealogy of AC populations.

### The asymmetric Wright–Fisher framework

Let us now focus on AWF models in which, in contrast to AC models, the reproduction dynamics of the individuals are given in terms of weights through a Bayesian-type parametrization, rather than in absolute number of children directly. This is a common mathematical strategy in the modeling of biological phenomena, which often yields tractable yet wide-encompassing models.

In this setting, ${\varvec{\xi }}^{(N)}(t)$ has multinomial distribution with parameters $(N,{\varvec{\eta }}^{(N)})$ where ${\varvec{\eta }}^{(N)}$ is a random probability vector. Children independently choose their parent in the previous generation according to ${\varvec{\eta }}^{(N)}$; and grouping children by their parent gives in fact the coagulation increment $\tilde{\Pi }^{(N)}_h$ appearing in (1). Indeed, grouping children in this way is precisely the well-known paint-box construction (see e.g. section 2.3.2 in Bertoin (2006)) of exchangeable random partitions directed by the mass partition ${\varvec{\eta }}^{(N)}$. By a simple “putting balls into boxes” argument analogous to that leading to (3) above, the transition matrix ${\varvec{P}}^{(N,n)}$ of $\left( \varPi ^{(N,n)}_t\right) _{t\in \mathbb {N}}$ is given in this case by

6 $$\begin{aligned} {\varvec{P}}^{(N,n)}(\tilde{\pi }) = \mathbbm {E}\left[ \sum _{\begin{array}{c} i_1,\dots ,i_{\left|\tilde{\pi } \right|} \\ \text {all distinct} \end{array}} \left( \eta ^{(N)}_{i_1}\right) ^{b_1} \cdots \left( \eta ^{(N)}_{i_{\left|\tilde{\pi } \right|}}\right) ^{b_{\left|\tilde{\pi } \right|}} \right] \end{aligned}$$

where $b_i{:=}\left|\tilde{\pi }_i \right|$, $1\le i\le \left|\tilde{\pi } \right|$. In particular, in the AWF setting, the probability that two randomly chosen individuals have the same parent in the previous generation is now

7 $$\begin{aligned} c_N = \mathbbm {E}\left[ \sum _{i=1}^N \left( \eta ^{(N)}_i\right) ^2\right] . \end{aligned}$$

Plugging these into (4), Theorem 2.1 yields a general criterion for the convergence of the genealogy of AWF populations.

We now give a characterization of (4) for AWF populations, in the same spirit as is done in Möhle and Sagitov (2001) for exchangeable offspring distributions. Our technique, and analogous results, should apply to more general cases. Let us introduce further notation. We call $(s_1,s_2,\dots )$ a size-biased reordering of a random probability vector ${\varvec{\eta }}^{(N)}=\left( \eta ^{(N)}_1,\dots ,\eta ^{(N)}_N\right)$ if, conditional on ${\varvec{\eta }}^{(N)}$ and letting $K=\sum _{j=1}^N \mathbbm {1}_{\eta ^{(N)}_j>0}$ be the number of non-zero entries of ${\varvec{\eta }}^{(N)}$, the vector $(s_1,\dots ,s_{K})$ is a size-biased reordering of the non-zero entries of ${\varvec{\eta }}^{(N)}$ in the usual sense (see e.g. Pitman and Yor (1997), or equation (9) below), and $s_j= 0$ for $j>K$.

#### Theorem 2.2

For an AWF population, the equality (4) holds for some $\Xi$-coalescent and $c_N\rightarrow 0$, i.e. case (ii) holds, if and only if the limits

8 $$\begin{aligned}&\phi _j(b_1,\dots ,b_j)\nonumber \\&= \lim _{N\rightarrow \infty } \frac{1}{c_N}\mathbbm {E}\left[ \prod _{i=1}^{j} \left( s^{(N)}_i\right) ^{b_i-1}\prod _{i=1}^{j-1} \left( 1-\sum _{k=1}^is^{(N)}_k\right) \right] \end{aligned}$$

exist for all $j\in \mathbb {N}$ and $b_1\ge \dots b_j\ge 2$, where $\left( s^{(N)}_1,s^{(N)}_2,\dots \right)$ is a size-biased reordering of ${\varvec{\eta }}^{(N)}$.

#### Proof

Recall (6). Observe that if $\eta ^{(N)}_{i}=0$ then the corresponding terms in the right hand side are zero. Also, for a size-biased reordering of ${\varvec{\eta }}^{(N)}$ and for distinct indices $i_1,\dots ,i_b$ such that $\eta _{i_j}>0$ for all $1\le j\le b$, we have

9 $$\begin{aligned}&\mathbbm {P}\left( s^{(N)}_k=\eta ^{(N)}_{i_k}, 1\le k\le b\vert {\varvec{\eta }}^{(N)}\right) \nonumber \\&=\prod _{k=1}^b \mathbbm {P}\left( s^{(N)}_k=\eta ^{(N)}_{i_k} \vert {\varvec{\eta }}^{(N)}, s^{(N)}_1, \cdots , s^{(N)}_{k-1}\right) \nonumber \\&=\prod _{k=1}^b \frac{\eta ^{(N)}_{i_k}}{1-\sum _{j=1}^{k-1}\eta ^{(N)}_{i_j}}. \end{aligned}$$

Thus, writing $S^{(N)}_i=s^{(N)}_1+\dots +s^{(N)}_i$, and $(b_1,\cdots ,b_{\left|\tilde{\pi } \right|})$ for the sizes of the blocks of $\tilde{\pi }$ arranged in non-increasing order,

$$\begin{aligned}&{\varvec{P}}^{(N,n)}({\pi ,{{\,\textrm{Coag}\,}}(\pi , \tilde{\pi })})\\ &=\sum _{ \begin{array}{c} i_1,\dots ,i_{\left|\tilde{\pi } \right|} \\ \text {all distinct} \end{array}} \mathbbm {E}\left[ \left( \eta ^{(N)}_{i_1}\mathbbm {1}_{\eta ^{(N)}_{i_1}>0}\right) ^{b_1} \cdots \left( \eta ^{(N)}_{i_{\left|\tilde{\pi } \right|}}\mathbbm {1}_{\eta ^{(N)}_{i_{\left|\tilde{\pi } \right|}}>0}\right) ^{b_{\left|\tilde{\pi } \right|}} \right] \\&=\sum _{ \begin{array}{c} i_1,\dots ,i_{\left|\tilde{\pi } \right|} \\ \text {all distinct} \end{array}} \mathbbm {E}\left[ \prod _{k=1}^{\left|\tilde{\pi } \right|} \frac{\eta ^{(N)}_{i_k}\mathbbm {1}_{\eta ^{(N)}_{i_k}>0}}{1-\sum _{j=1}^{k-1}\eta ^{(N)}_{i_j}} (1-\sum _{j=1}^{k-1}\eta ^{(N)}_{i_j})\left( \eta ^{(N)}_{i_k}\right) ^{b_k-1} \right] \\&=\mathbbm {E}\left[ \sum _{ \begin{array}{c} i_1,\dots ,i_{\left|\tilde{\pi } \right|} \\ \text {all distinct} \end{array}} \mathbbm {P}\left( s^{(N)}_k=\eta ^{(N)}_{i_k}, k \le \left|\tilde{\pi } \right| \vert {\varvec{\eta }}^{(N)}\right) \prod _{i=1}^{\left|\tilde{\pi } \right|} (1-\sum _{j=1}^{k-1}\eta ^{(N)}_{i_j})\left( \eta ^{(N)}_{i_k}\right) ^{b_k-1} \right] \\&=\mathbbm {E}\left[ \mathbbm {E}\left[ \prod _{i=1}^{\left|\tilde{\pi } \right|}\left( s^{(N)}_{i}\right) ^{b_i-1} \prod _{i=1}^{\left|\tilde{\pi } \right|-1} (1-S^{(N)}_i); s^{(N)}_1>0, \dots , s^{(N)}_{\left|\tilde{\pi } \right|}>0 \vert {\varvec{\eta }}^{(N)}\right] \right] \\&=\mathbbm {E}\left[ \prod _{i=1}^{\left|\tilde{\pi } \right|}\left( s^{(N)}_{i}\right) ^{b_i-1} \prod _{i=1}^{\left|\tilde{\pi } \right|-1} (1-S^{(N)}_i); s^{(N)}_1>0, \dots , s^{(N)}_{\left|\tilde{\pi } \right|}>0 \right] . \end{aligned}$$

Note that the r.h.s. above does not depend on $\pi$ and that (depending only on its block sizes) it is invariant under permutations of the partition $\tilde{\pi }$, i.e. for any finite permutation $\sigma$ we have

10 $$\begin{aligned} {\varvec{P}}^{(N,n)}({\pi ,{{\,\textrm{Coag}\,}}(\pi , \tilde{\pi })}) ={\varvec{P}}^{(N,n)}({\pi ,{{\,\textrm{Coag}\,}}(\pi , \sigma (\tilde{\pi }))}). \end{aligned}$$

On the other hand, let us assume for the moment that (8) exists for all $j\ge 1$ and $b_1\ge \dots b_j\ge 1$. Then, we have the recursion,

11 $$\begin{aligned}&\phi (b_1,\dots ,b_j,1) + \phi (b_1+1,b_2,\dots ,b_j) + \cdots + \phi (b_1,\cdots ,b_j+1)\nonumber \\&\quad = \lim _{N\rightarrow \infty } \tfrac{\mathbbm {E}\left[ (S^{(N)}_j + (1-S^{(N)}_j))\left( \prod _{i=1}^{j} \left( s^{(N)}_i\right) ^{b_j-1}\right) \prod _{i=1}^{j-1} \left( 1-S^{(N)}_i\right) ; s^{(N)}_i>0, 1\le i\le j\right] }{c_N}\nonumber \\&\quad =\phi (b_1,\cdots ,b_j). \end{aligned}$$

Equations (10) and (11) (for all $b_1\ge \dots b_j\ge 1$) allow us to construct a measure $\mu$ on $\mathscr {P}_{\infty }$. For any $n\in \mathbb {N}$ and $\pi '\in \mathscr {P}_{\infty }$ let $\pi '\vert _n$ be the restriction of $\pi '$ to [*n*], and denote by $\mathscr {P}_{\infty }(\pi )$ the set $\mathscr {P}_{\infty }(\pi ){:=}\{\pi '\in \mathscr {P}_{\infty }:\pi '\vert _n=\pi \}$. Then, setting for any partition $\pi \in \mathscr {P}_{n}$ with ordered block sizes $(b_1,\cdots ,b_{\left|\pi \right|})$,

12 $$\begin{aligned} \mu \left( \mathscr {P}_{\infty }(\pi )\right) = \phi _{\left|\pi \right|}(b_1,\dots ,b_{\left|\pi \right|}) \end{aligned}$$

defines a measure on $\mathscr {P}_{\infty }$.

Indeed, the measure $\mu$ can be formally constructed via Caratheodory’s extension theorem applied to the measure ${\hat{\mu }}$ on the semi-ring $\mathscr {S}{:=}\{\mathscr {P}_{\infty }(\pi ): \pi \in \mathscr {P}_{n}{\setminus }\textbf{0}_n, \hspace{6pt} n\in \mathbb {N}\}$ defined analogously through (12). The finite-additivity of ${\hat{\mu }}$ follows easily from (12). Infinite-additivity follows from the fact that $\mathscr {P}_{\infty }$ is compact (see Lemma 2.6 in Bertoin (2006)) and the fact that the elements of $\mathscr {S}$ are both open and closed. Indeed, the latter implies that if $\mathscr {S}\ni A = \cup _{i=1}^\infty A_i$, $A_i \in \mathscr {S}$, then, in fact, there exists a finite collection of indices $i_1,\dots ,i_k$ such that $A=\cup _{j=1}^k A_{i_j}$. Thus, infinite-additivity follows from finite-additivity. The measure $\mu$ being invariant under finite permutations (by construction), Theorem 4.2 in Bertoin (2006) ensures the existence of a measure $\Xi$ on $\mathscr {P}_{[0,1]}$ such that

$$\begin{aligned} \mu (\pi )={\varvec{Q}}^{(n)}_{\Xi }(\pi ),\quad \forall n\in \mathbb {N}, \pi \in \mathscr {P}_{n}\setminus \textbf{0}_n. \end{aligned}$$

Finally, it only remains to note that in fact it is enough to verify (8) for block sizes satisfying $b_1\ge b_2\ge \dots \ge b_j\ge 2$. In other words, we need to show that in this case the limit in (8) will also exist for the greater set of block sizes $b_1\ge b_2\ge \dots \ge b_j\ge 1$. The latter follows from the recursion in (11) which yields that if (8) exists for $b_1\ge b_2\ge \dots \ge b_j\ge 2$ then it also must exist for $b_1\ge b_2\ge \dots \ge b_j\ge 2, b_{j+1}=1$, which through a second call to (11), entails that (8) must also exist for $b_1\ge b_2\ge \dots \ge b_j\ge 2, b_{j+1}=1, b_{j+2}=1$, and so forth. $\square$

### From AC to AWF models

The main result of this section says that, in most cases, the sampling (of the parents) with and without replacement become equivalent in the limit when $N\rightarrow \infty$; so that the genealogies of asymmetric neutral populations can be mostly studied by the results described so far for AWF populations. In the same spirit as in Schweinsberg (2003), let us consider an extension of our current setting. We assume that the total offspring size $\Sigma _N=\sum _{i=1}^N \xi _{i}^{(N)}$ satisfies

13 $$\begin{aligned} \Sigma _N\ge N \end{aligned}$$

almost surely and, to form the next generation, a sample without replacement of size *N* among the newly produced children is taken. Our next proposition is inspired by and generalizes the relationship between the models of Schweinsberg (2003) and Huillet and Möhle (2021).

#### Proposition 2.3

Let $\left( \xi _{1}^{(N)},\dots ,\xi _{N}^{(N)}\right)$ be a random vector of non-negative integers such that (13) holds. Let $\tilde{{\varvec{P}}}^{(N,n)}$ be the corresponding coagulation probabilities for a sample of *n* individuals: for any $\tilde{\pi }\in \mathscr {P}_{n}$,

$$\begin{aligned} \tilde{{\varvec{P}}}^{(N,n)}(\tilde{\pi }) = \mathbbm {E}\left[ \sum _{\begin{array}{c} i_1,\dots ,i_{\left|\tilde{\pi } \right|} \\ \text {all distinct} \end{array}} \frac{\left( \xi _{i_1}^{(N)}\right) _{b_1} \cdots \left( \xi _{i_{\left|\tilde{\pi } \right|}}^{(N)}\right) _{b_{\left|\tilde{\pi } \right|}}}{(\Sigma _N)_{n}} \right] . \end{aligned}$$

Also let

14 $$\begin{aligned} {\tilde{c}}_N{:=}\tilde{{\varvec{P}}}^{(N,2)}(\{\{1,2\}\})\equiv \mathbbm {E}\left[ \sum _{i=1}^N \frac{\xi _{i}^{(N)}\left( \xi _{i}^{(N)}-1\right) }{\Sigma _N(\Sigma _N-1)} \right] . \end{aligned}$$

Set $\eta ^{(N)}_i={\xi _{i}^{(N)}}/{\Sigma _N}$ and consider the corresponding AWF population with coagulation probabilities ${{\varvec{P}}}^{(N,n)}$ as in (6) and $c_N$ as in (7). Then for every $n\ge 2$ there exists $C(n)>0$ such that, for sufficiently large *N* and in the usual operator norm

15 $$\begin{aligned} \left\Vert \tilde{{\varvec{P}}}^{(N,n)} - {{\varvec{P}}}^{(N,n)} \right\Vert \le C(n)\mathbbm {E}\left[ \frac{1}{\Sigma _N}\right] . \end{aligned}$$

In particular, whenever either $c_N/\mathbbm {E}\left[ {1}/{\Sigma _N}\right] \rightarrow \infty$ or ${\tilde{c}}_N/\mathbbm {E}\left[ {1}/{\Sigma _N}\right] \rightarrow \infty$ as $N\rightarrow \infty$, we have $c_N\sim {\tilde{c}}_N$ and, furthermore, both models have the same limit genealogy in distribution.

#### Remark

Many examples in the literature, in particular those whose limit genealogies are not described by Kingman’s coalescent, satisfy either $\Sigma _N/N\rightarrow \infty$ or $c_NN\rightarrow \infty$, both of which imply the condition $c_N/\mathbbm {E}\left[ {1}/{\Sigma _N}\right] \rightarrow \infty$. (For instance in Schweinsberg (2003) and Huillet and Möhle (2021), where Beta coalescents appear, one has $c_N=\mathcal {O}\left( N^{1-\alpha }\right)$ with $\alpha \in (1,2)$; but see also Cortines and Mallein (2017) and Sect. 5 below). However, Proposition 2.3 cannot be used to show the well known equivalence between some classical models in which $c_N\sim 1/N$. For example between the classical Wright–Fisher model $(\eta ^{(N)}_i=1/N)$ and the corresponding Cannings model where ${\varvec{\xi }}^{(N)}$ has multinomial distribution of parameter $(p_i=1/N)_{1\le i\le N}$. Both of these models converge to Kingman’s coalescent.

#### Proof

Stirling’s approximation in the form $\Gamma (m+a)/\Gamma (m+b)=m^{a-b}(1+\mathcal {O}\left( 1/m\right) )$ as $m\rightarrow \infty$, and the condition (13) yield, for some constant $C\equiv C(n)$ and sufficiently large *N*, the almost sure inequalities

$$\begin{aligned} \Sigma _N^n\left( 1-C/\Sigma _N\right) {\le } (\Sigma _N)_n {\le }\Sigma _N^n\left( 1+C/\Sigma _N\right) , \end{aligned}$$

so that

$$\begin{aligned} \frac{1}{ (\Sigma _N)_n}&\ge \frac{1}{\Sigma _N^n} + \frac{1}{\Sigma _N^n} \left( \frac{1}{1+C/\Sigma _N} - 1\right) \\&\ge \frac{1}{\Sigma _N^n} + \frac{1}{\Sigma _N^n} \left( \frac{C}{\Sigma _N}\right) \end{aligned}$$

and

$$\begin{aligned} \frac{1}{ (\Sigma _N)_n}&\le \frac{1}{\Sigma _N^n} + \frac{1}{\Sigma _N^n} \left( \frac{1}{1-C/\Sigma _N} - 1\right) \\&\le \frac{1}{\Sigma _N^n} + \frac{1}{\Sigma _N^n} \left( \frac{C}{\Sigma _N}\right) . \end{aligned}$$

Thus

$$\begin{aligned} \tilde{{\varvec{P}}}^{(N,n)} (\tilde{\pi }) = \mathbbm {E}\left[ \sum _{\begin{array}{c} i_1,\dots ,i_{\left|\tilde{\pi } \right|} \\ \text {all distinct} \end{array}} \frac{\left( \xi _{i_1}^{(N)}\right) _{b_1} \cdots \left( \xi _{i_{\left|\tilde{\pi } \right|}}^{(N)}\right) _{b_{\left|\tilde{\pi } \right|}}}{\Sigma _N^{n}} \right] + \left( \mathbbm {E}\left[ \frac{1}{\Sigma _N}\right] \right) . \end{aligned}$$

To deal with the numerators in the sum above, first the bound $(a)_b =a(a-1)\cdots (a-b+1)\le a^b,$ already yields

$$\begin{aligned} \tilde{{\varvec{P}}}^{(N,n)} (\tilde{\pi }) \le \mathbbm {E}\left[ \sum _{\begin{array}{c} i_1,\dots ,i_{\left|\tilde{\pi } \right|} \\ \text {all distinct} \end{array}} \frac{\left( \xi _{i_1}^{(N)}\right) ^{b_1} \cdots \left( \xi _{i_{\left|\tilde{\pi } \right|}}^{(N)}\right) ^{b_{\left|\tilde{\pi } \right|}}}{\Sigma _N^{n}} \right] + \left( \mathbbm {E}\left[ \frac{1}{\Sigma _N}\right] \right) , \end{aligned}$$

where the first term in the r.h.s is just the expression for the coagulation probabilities ${{\varvec{P}}}^{(N,n)}$ of the AWF population. We now prove the corresponding lower bound. Using that for $x\in [0,1]$, $(1-x)^b-1{\ge -bx}$ we obtain, for $a>b-1$,

$$\begin{aligned} (a)_b\ge (a-b+1)^b {\ge a^b - a^{b-1}b(b-1)}. \end{aligned}$$

Then for any $1\le j\le \ell \le n$ and $n\ge b_1\ge \cdots \ge b_j\ge 2, b_{j+1}=\cdots =b_{\ell }=1$ we have, writing $D_{i,b}{:=}\left( \left( \eta ^{(N)}_{i}\right) ^{b} - \frac{\left( \eta ^{(N)}_{i}\right) ^{b-1}}{{\Sigma _N}}b(b{-}1)\right) \mathbbm {1}_{\xi _{i}^{(N)}>b-1}$ and setting empty products to 1,

16 $$\begin{aligned}&\sum _{\begin{array}{c} i_1,\dots ,i_{\ell } \\ \text {all distinct} \end{array}} \frac{\left( \xi _{i_1}^{(N)}\right) _{b_1}}{\Sigma _N^{b_1}} \cdots \frac{\left( \xi _{i_{j}}^{(N)}\right) _{b_{j}}}{\Sigma _N^{b_j}} \prod _{k=j+1}^\ell \eta ^{(N)}_{i_k}\nonumber \\&\quad \ge \sum _{\begin{array}{c} i_1,\dots ,i_{\ell } \\ \text {all distinct} \end{array}} \prod _{k=1}^j D_{i_k,b_k}\prod _{k=j+1}^\ell \eta ^{(N)}_{i_k}\mathbbm {1}_{\xi _{i_k}^{(N)}\ge 1} \\&\quad = \sum _{\begin{array}{c} i_1,\dots ,i_{\ell } \\ \text {all distinct} \end{array}} \left( \eta ^{(N)}_{i_1}\right) ^{b_1} \mathbbm {1}_{\xi _{i_1}^{(N)}>b_1-1} \prod _{k=2}^j D_{i_k,b_k} \prod _{k=j+1}^\ell \eta ^{(N)}_{i_k}\mathbbm {1}_{\xi _{i_k}^{(N)}\ge 1}\nonumber \\&\qquad - b_1(b_1-1) \sum _{i_1=1}^N \frac{\left( \eta ^{(N)}_{i_1}\right) ^{b_1-1}}{\Sigma _N}\mathbbm {1}_{\xi _{i_1}^{(N)}>b_1-1}\nonumber \\&\sum _{\begin{array}{c} i_2,\dots ,i_{\ell }\subset [N]\setminus \{i_1\} \\ \text {all distinct} \end{array}} \prod _{k=2}^j D_{i_k,b_k} \prod _{k=j+1}^\ell \eta ^{(N)}_{i_k}\mathbbm {1}_{\xi _{i_k}^{(N)}\ge 1} .\nonumber \end{aligned}$$

Since $\sum _{i=1}^N \eta ^{(N)}_i = 1$, we obtain the following bound for the second term above

17 $$\begin{aligned}&\sum _{i_1=1}^N \frac{\left( \eta ^{(N)}_{i_1}\right) ^{b_1-1}}{\Sigma _N}\mathbbm {1}_{\xi _{i_1}^{(N)}>b_1-1}\sum _{\begin{array}{c} i_2,\dots ,i_{\ell }\subset [N]\setminus \{i_1\} \\ \text {all distinct} \end{array}} \prod _{k=2}^j D_{i_k,b_k} \prod _{k=j+1}^\ell \eta ^{(N)}_{i_k}\mathbbm {1}_{\xi _{i_k}^{(N)}\ge 1}\nonumber \\&\le \frac{1}{\Sigma _N} \sum _{\begin{array}{c} i_1,\dots ,i_{\ell } \\ \text {all distinct} \end{array}} \eta ^{(N)}_{i_1}\cdots \eta ^{(N)}_{i_\ell }\le \frac{1}{\Sigma _N}. \end{aligned}$$

Iterating over $\{1,\dots ,j\}$ and plugging the corresponding bounds (17) into (16) we obtain, for some $C(b_1,\dots ,b_j)\equiv C >0$,

18 $$\begin{aligned}&\mathbbm {E}\left[ \sum _{\begin{array}{c} i_1,\dots ,i_{\ell }\\ \text {all distinct} \end{array}} \frac{\left( \xi _{i_1}^{(N)}\right) _{b_1}}{\Sigma _N^{b_1}} \cdots \frac{\left( \xi _{i_{j}}^{(N)}\right) _{b_{j}}}{\Sigma _N^{b_j}} \prod _{k=j+1}^\ell \eta ^{(N)}_{i_k} \right] \nonumber \\&\quad \ge \mathbbm {E}\left[ \sum _{\begin{array}{c} i_1,\dots ,i_{\ell } \\ \text {all distinct} \end{array}} \prod _{k=1}^j\left( \eta ^{(N)}_{i_k}\right) ^{b_k} \mathbbm {1}_{ \xi _{i_k}^{(N)}>b_k-1}\prod _{k=j+1}^\ell \eta ^{(N)}_{i_k} \right] - \mathbbm {E}\left[ \frac{C}{\Sigma _N}\right] . \end{aligned}$$

Now to “remove” the indicator functions appearing inside the sum above, observe that

19 $$\begin{aligned}&\mathbbm {E}\left[ \sum _{\begin{array}{c} i_1,\dots ,i_{\ell } \\ \text {all distinct} \end{array}} \left( \eta ^{(N)}_{i_1}\right) ^{b_1} \cdots \left( \eta ^{(N)}_{i_\ell }\right) ^{b_\ell } \mathbbm {1}_{\cup _{k=1}^j \{\xi _{i_k}^{(N)}\le b_k-1\}} \right] \nonumber \\&\le \mathbbm {E}\left[ \sum _{\begin{array}{c} i_1,\dots ,i_{\ell } \\ \text {all distinct} \end{array}} \left( \eta ^{(N)}_{i_1}\right) ^{b_1} \cdots \left( \eta ^{(N)}_{i_\ell }\right) ^{b_\ell } \sum _{k=1}^j \mathbbm {1}_{\xi _{i_k}^{(N)}\le b_k-1} \right] \\&\le \sum _{k=1}^j \mathbbm {E}\left[ \sum _{\begin{array}{c} i_1,\dots ,i_{\ell } \\ \text {all distinct} \end{array}} \left( \eta ^{(N)}_{i_1}\right) ^{b_1} \cdots \left( \eta ^{(N)}_{i_{k-1}}\right) ^{b_{k-1}} \left( \frac{b_k-1}{\Sigma _N}\right) ^{b_k} \left( \eta ^{(N)}_{i_{k+1}}\right) ^{b_{k+1}} \cdots \left( \eta ^{(N)}_{i_j}\right) ^{b_{j}} \right] \nonumber \\&\le \sum _{k=1}^j \mathbbm {E}\left[ \left( \frac{b_k-1}{\Sigma _N}\right) ^{b_k} \sum _{\begin{array}{c} i_1,\dots ,i_{\ell -1} \\ \text {all distinct} \end{array}} \left( \eta ^{(N)}_{i_1}\right) ^{b_1} \cdots \left( \eta ^{(N)}_{i_{\ell -1}}\right) ^{b_{\ell -1}} \right] \le \sum _{k=1}^j \mathbbm {E}\left[ \left( \frac{b_k-1}{\Sigma _N}\right) ^{b_k}\right] .\nonumber \end{aligned}$$

Since $n\ge b_1\ge b_\ell \ge 1$ and $j\le \ell \le n$, the last expression becomes bounded by $n(n-1)^{n}\mathbbm {E}\left[ \Sigma _N^{-1}\right]$. Thus, adding and subtracting (19) into (18), we obtain for some $C(b_1,\dots ,b_\ell )\equiv C'>0$,

20 $$\begin{aligned}&\mathbbm {E}\left[ \sum _{\begin{array}{c} i_1,\dots ,i_{\ell } \\ \text {all distinct} \end{array}} \frac{\left( \xi _{i_1}^{(N)}\right) _{b_1}}{\Sigma _N^{b_1}} \cdots \frac{\left( \xi _{i_{j}}^{(N)}\right) _{b_{j}}}{\Sigma _N^{b_j}} \prod _{k=j+1}^\ell \eta ^{(N)}_{i_k} \right] \nonumber \\&\ge \mathbbm {E}\left[ \sum _{\begin{array}{c} i_1,\dots ,i_{\ell } \\ \text {all distinct} \end{array}} \left( \eta ^{(N)}_{i_1}\right) ^{b_1} \cdots \left( \eta ^{(N)}_{i_\ell }\right) ^{b_\ell } \right] - \mathbbm {E}\left[ \frac{C'}{\Sigma _N}\right] . \end{aligned}$$

The last statement of the proposition follows from observing that if $c_N/\mathbbm {E}\left[ \Sigma _N^{-1}\right] \rightarrow \infty$ as $N\rightarrow \infty$ then $\mathbbm {E}\left[ \Sigma _N^{-1}\right] =o\big (c_N\big )$ and, furthermore, equation (15) (with $n=2$) yields $c_N\sim {\tilde{c}}_N$. Thus

$$\begin{aligned} \left\Vert \tilde{{\varvec{P}}}^{(N,n)} - {{\varvec{P}}}^{(N,n)} \right\Vert =o\big (c_N\big )=o\big ({\tilde{c}}_N\big ). \end{aligned}$$

The latter implies ${\varvec{P}}^{(N,n)}(\tilde{\pi }) = c_N{\varvec{Q}}^{(n)}_{\Xi }(\tilde{\pi }) + o\big (c_N\big )$ if and only if $\tilde{{\varvec{P}}}^{(N,n)}(\tilde{\pi }) = {\tilde{c}}_N{\varvec{Q}}^{(n)}_{\Xi }(\tilde{\pi }) + o\big ({\tilde{c}}_N\big )$ so that, by Theorem 2.1, the limit coalescents of both models coincide. $\square$

Let us end this section with a simple application of the above theorem.

#### Corollary 2.4

In the setting of Proposition 2.3, let ${\varvec{\rho }}^{(N)}$ be the vector ${\varvec{\eta }}^{(N)}$ rearranged in decreasing order. Assume that ${\varvec{\rho }}^{(N)}$ converges in distribution in $\mathscr {P}_{[0,1]}$ to ${\varvec{\rho }}^{(\infty )}$ as $N\rightarrow \infty$. Then the genealogies of both the AWF and the corresponding AC models converge in distribution in the product topology for $\left( \mathscr {P}_{n}\right) ^\mathbb {N}$ to a Markov chain with initial state $\textbf{0}_n$ and transition matrix given by

$$\begin{aligned} {\varvec{P}}^{(\infty ,n)}(\pi , {{\,\textrm{Coag}\,}}(\pi , \tilde{\pi })) = \mathbbm {P}\left( \tilde{\Pi }^{(\infty ,n)} = \tilde{\pi }\right) , \end{aligned}$$

where $\tilde{\Pi }^{(\infty ,n)}\in \mathscr {P}_{n}$ is obtained through the paintbox construction from ${\varvec{\rho }}^{(\infty )}$.

#### Proof

Since $c_N\rightarrow \mathbbm {E}[\sum _{i=1}^\infty (\rho ^{(\infty )}_i)^2]>0$ and $\Sigma _N=N$, by Proposition 2.3 we need only proof the result for the AWF model. Let $\tilde{\Pi }^{(N,n)}\in \mathscr {P}_{n}$ be a paint-box constructed from ${\varvec{\rho }}^{(N)}$. Proposition 2.9 in Bertoin (2006) readily gives, for any $\tilde{\pi }\in \mathscr {P}_{n}$,

$$\begin{aligned} \mathbbm {P}\left( \tilde{\Pi }^{(N,n)} = \tilde{\pi }\right) = \mathbbm {P}\left( \tilde{\Pi }^{(\infty ,n)} = \tilde{\pi }\right) + o\big (1\big ), \end{aligned}$$

which yields the condition of Theorem 2.1 for the case $\lim _{N\rightarrow \infty }c_N>0$ with $c=\mathbbm {E}[\sum _{i=1}^\infty (\rho ^{(\infty )}_i)^2]$. $\square$

## Simpler criteria for convergence to $\Lambda$-coalescents

In this section we provide a simpler sufficient condition for (4) to hold for AWF models whose genealogies converge to a $\Lambda$-coalescent (Pitman 1999; Sagitov 1999). A $\Lambda$-coalescent corresponds to the case in which the coagulation measure $\Xi$ is supported on mass partitions of the form $(p,0,0,\cdots )$, and we denote by $\Lambda$ the pushforward of $\Xi$ after projecting into the first coordinate $p\in (0,1)$. In particular, the partition-valued process evolves through simple coaugulations, in which a single group of *b* blocks merge together at rate

$$\begin{aligned} \lambda _{n,b}{:=}\int _0^1 p^{b-2}(1-p)^{n-b} \Lambda (dp) \end{aligned}$$

whenever there are *n* blocks in the system.

There are two simple heuristics from distinct population dynamics which motivate this section (and the appearance of $\Lambda$-coalescents in general): 1) all the individuals in the population have the same fitness but occasionally, by mere chance, there occur large reproductive events where a single individual takes up a large fraction of the offspring in the next generation, and 2) the offspring distribution is highly asymmetric and the family frequencies given by the mass-partition ${\varvec{\eta }}^{(N)}$ are dominated by the largest family frequency (say $\rho ^{(N)}_1$) associated to the individual with the highest reproductive success. In both of these scenarios we expect that all the coagulations that remain observable in the limit correspond to very large reproduction events of single individuals along the generations (see Schweinsberg 2003; Huillet and Möhle 2021; Cortines and Mallein 2017); these coagulations must then be driven by $\rho ^{(N)}_1$. In practice we would rather allow more flexibility and prove that all the coagulations that “survive” in the limit are driven by the frequency $\eta ^{(N)}_{1}$. This entails that $\eta ^{(N)}_{1}$ should approximate $\rho ^{(N)}_1$ as $N\rightarrow \infty$, but working with $\eta ^{(N)}_{1}$ instead of $\rho ^{(N)}_1$ may ease the study of the underlying genealogy of the population (also note that, the distribution of ${\varvec{\eta }}^{(N)}$ having no restrictions, our setup does include the case where $\eta ^{(N)}_1=\rho ^{(N)}_1$). Of particular interest is the case where $\left( \eta ^{(N)}_{1},\dots ,\eta ^{(N)}_{N}\right)$ is a (normalized) size-biased pick from a random mass partition for which the distribution may be explicitly known (see Sect. 5 for a more general example of this).

The first result, an easy consequence of Theorem 2.1, reads as follows.

### Proposition 3.1

Assume that there exists $i\in [N]$ such that, for every $b\ge 2$,

21 $$\begin{aligned} \mathbbm {E}\left[ \left( \eta ^{(N)}_i\right) ^b\right] \sim c_N \frac{ \lambda _{b,b}}{\lambda _{2,2}}. \end{aligned}$$

Then the genealogy $\big (\varPi ^{(N,n)}_{\lfloor t/c_N\rfloor }\big )_{t\ge 0}$ of the corresponding AWF population converges weakly in the Skorohod space $D([0,\infty ), \mathscr {P}_{n})$ to a coalescent with characteristic measure $\Lambda$.

### Proof

With no loss of generality, suppose that $i=1$. The definition of $c_N$ in (7), plus the hypothesis (21) when $b=2$ give

22 $$\begin{aligned} \mathbbm {E}\left[ \sum _{i=2}^N\left( \eta ^{(N)}_i\right) ^2\right] = o\big (c_N\big ). \end{aligned}$$

Also note that, for the sum appearing in (6) but restricted to indices greater than 2, we have

23 $$\begin{aligned}&\sum _{\begin{array}{c} i_1,i_2,\dots ,i_j \ge 2\\ \text {all distinct} \end{array}} \left( \eta ^{(N)}_{i_1}\right) ^{b_1}\cdots \left( \eta ^{(N)}_{i_j}\right) ^{b_j}\nonumber \\ &\quad = \sum _{\begin{array}{c} i_1,i_2,\dots ,i_{j-1}\ge 2 \\ \text {all distinct} \end{array}} \left( \eta ^{(N)}_{i_1}\right) ^{b_1}\cdots \left( \eta ^{(N)}_{i_{j-1}}\right) ^{b_{j-1}} \sum _{i_j\not \in \{i_1,\dots ,i_{j-1}\}} \left( \eta ^{(N)}_{i_j}\right) ^{b_j}\nonumber \\ &\quad \le \sum _{\begin{array}{c} i_1,i_2\dots ,i_{j-1}\ge 2 \\ \text {all distinct} \end{array}} \left( \eta ^{(N)}_{i_1}\right) ^{b_1}\cdots \left( \eta ^{(N)}_{i_{j-1}}\right) ^{b_{j-1}} \nonumber \\ &\quad \vdots \nonumber \\ &\quad \le \sum _{i=2}^N \left( \eta ^{(N)}_{i}\right) ^{b_1}\le \sum _{i=2}^N \left( \eta ^{(N)}_{i}\right) ^{2}. \end{aligned}$$

The latter implies, for any $\pi \in \mathscr {P}_{n}$ and coagulation increment $\tilde{\pi }\in \mathscr {P}_{\left|\pi \right|}\setminus \textbf{0}_{\left|\pi \right|}$ with ordered block sizes $b_1\ge \dots \ge b_{j}$, $j=\left|\tilde{\pi } \right|$,

$$\begin{aligned} {{\varvec{P}}}^{(N,n)}({\pi ,{{\,\textrm{Coag}\,}}\left( \pi ,\tilde{\pi }\right) })&= \mathbbm {E}\left[ \sum _{\begin{array}{c} i_1=1,i_2,\dots ,i_{j}\\ \text {all distinct} \end{array}} \left( \eta ^{(N)}_{i_1}\right) ^{b_1}\cdots \left( \eta ^{(N)}_{i_j}\right) ^{b_j}\right] + o\big (c_N\big )\\&=\mathbbm {E}\left[ \left( \eta ^{(N)}_{1}\right) ^{b_1} \sum _{\begin{array}{c} i_2,\dots ,i_j\ge 2 \\ \text {all distinct} \end{array}} \left( \eta ^{(N)}_{i_2}\right) ^{b_2}\cdots \left( \eta ^{(N)}_{i_j}\right) ^{b_j}\right] +o\big (c_N\big ). \end{aligned}$$

Again, using (23) and (22), we have

$$\begin{aligned}&\mathbbm {E}\left[ \left( \eta ^{(N)}_{1}\right) ^{b_1}\sum _{\begin{array}{c} i_2,\dots ,i_j\ge 2\\ \text {all distinct} \end{array}} \left( \eta ^{(N)}_{i_2}\right) ^{b_2}\cdots \left( \eta ^{(N)}_{i_j}\right) ^{b_j}\right] \\&\quad = {\left\{ \begin{array}{ll} o\big (c_N\big ) & \text { if }b_2\ge 2,\\ \mathbbm {E}\left[ \left( \eta ^{(N)}_{1}\right) ^{b_1}(1-\eta ^{(N)}_{1})^{\left|\pi \right|-b_1}\right] & \text { otherwise}. \end{array}\right. } \end{aligned}$$

It thus remains to prove that

24 $$\begin{aligned} \mathbbm {E}\left[ \left( \eta ^{(N)}_{1}\right) ^{b_1}(1-\eta ^{(N)}_{1})^{\left|\pi \right|-b_1}\right] \sim c_N\frac{\lambda _{\left|\pi \right|,b_1}}{\lambda _{2,2}} \end{aligned}$$

whenever $\left|\pi \right|\ge b_1\ge 2$. This is true when $\left|\pi \right|= b_1$, thanks to the assumption (21). This can be shown in whole generality thanks to an induction over the values of $\left|\pi \right|- b_1\ge 0$. Given the well-known recursion formula $\lambda _{\left|\pi \right|,b_1}=\lambda _{\left|\pi \right|+1,b_1}+\lambda _{\left|\pi \right|+1,b_1+1}$ (Lemma 18 Pitman (1999)) on the one side, and the recursion

$$\begin{aligned}&\mathbbm {E}\left[ \left( \eta ^{(N)}_{1}\right) ^{b_1}(1-\eta ^{(N)}_{1})^{\left|\pi \right|-b_1}\right] = \mathbbm {E}\left[ \left( \eta ^{(N)}_{1}\right) ^{b_1}(1-\eta ^{(N)}_{1})^{\left|\pi \right|+1-b_1}\right] \\&\quad + \mathbbm {E}\left[ \left( \eta ^{(N)}_{1}\right) ^{b_1+1}(1-\eta ^{(N)}_{1})^{\left|\pi \right|+1-b_1-1}\right] , \end{aligned}$$

on the other, it is not difficult to see that (24) for any choice of $\left|\pi \right|- b_1=k\ge 0$ implies the same equivalence for the cases $\left|\pi \right|-b_1=k+1$. $\square$

The above proposition provides a mild improvement to Lemma 3.1 in Cortines and Mallein (2017) since, in our case, the family frequencies need not to be arranged in decreasing order. We now present our simplified criterion for the convergence to Kingman’s coalescent which is in correspondence with their Lemma 3.2. Note that, in this case, and as is common in scenarios with convergence to Kingman’s coalescent, it is no longer the case that the coagulation events that remain in the limit are driven by a single family frequency $\eta ^{(N)}_i$.

### Proposition 3.2

Assume that

$$\begin{aligned} \mathbbm {E}\left[ \left( \eta ^{(N)}_2\right) ^3 + \dots + \left( \eta ^{(N)}_N\right) ^3\right] = o\big (c_N\big ), \end{aligned}$$

and that there exists $\beta >2$ such that

$$\begin{aligned} \mathbbm {E}\left[ \left( \eta ^{(N)}_1\right) ^\beta \right] = o\big (c_N\big ). \end{aligned}$$

Then

25 $$\begin{aligned} \mathbbm {E}\left[ \left( \eta ^{(N)}_1\right) ^3 + \dots + \left( \eta ^{(N)}_N\right) ^3\right] = o\big (c_N\big ). \end{aligned}$$

If, in addition, $c_N\rightarrow 0$, then the genealogy $\big (\varPi ^{(N,n)}_{\lfloor t/c_N\rfloor }\big )_{t\ge 0}$ of the corresponding AWF population converges weakly in the Skorohod topology for $D([0,\infty ), \mathscr {P}_{n})$ to Kingman’s coalescent.

### Proof

We first prove that the second hypothesis implies

$$\begin{aligned} \mathbbm {E}\left[ \left( \eta ^{(N)}_1\right) ^3\right] =o\big (c_N\big ). \end{aligned}$$

We follow an argument of Cortines and Mallein (2017). The result is trivial if $\beta \le 3$, we thus assume $\beta >3$. Observe that for $\lambda \in (0,2)$ we have, using Hölder’s inequality,

$$\begin{aligned} \mathbbm {E}\left[ \left( \eta ^{(N)}_{1}\right) ^{3}\right]&= \mathbbm {E}\left[ \left( \eta ^{(N)}_{1}\right) ^{\lambda }\left( \eta ^{(N)}_{1}\right) ^{3-\lambda }\right] \\&\le \mathbbm {E}\left[ \left( \eta ^{(N)}_{1}\right) ^2\right] ^{\lambda /2}\mathbbm {E}\left[ \left( \eta ^{(N)}_{1}\right) ^{\frac{2(3-\lambda )}{2-\lambda }}\right] ^{1-\lambda /2} \end{aligned}$$

so that, choosing $\lambda \in (0,2)$ to ensure $\frac{2(3-\lambda )}{2-\lambda }>\beta$, we obtain by the second hypothesis,

$$\begin{aligned} \frac{\mathbbm {E}\left[ \left( \eta ^{(N)}_{1}\right) ^{3}\right] }{c_N}&\le \frac{\mathbbm {E}\left[ \left( \eta ^{(N)}_{1}\right) ^{\frac{2(3-\lambda )}{2-\lambda }}\right] ^{1-\lambda /2}}{c_N^{1-\lambda /2}}\\&\le \left( c^{-1}_N\mathbbm {E}\left[ \left( \eta ^{(N)}_{1}\right) ^{\beta }\right] \right) ^{1-\lambda /2}, \end{aligned}$$

converging to 0 when ${N\rightarrow \infty }$. Combining with the first hypothesis, we conclude (25).

Now, let $b_1\ge \cdots b_j\ge 1$ be the ordered block sizes of the coagulation increment $\pi '$ in (6). By a similar computation as that in (23) we have

$$\begin{aligned} \sum _{\begin{array}{c} i_1,i_2,\dots ,i_j \nonumber \\ \text {all distinct} \end{array}} \left( \eta ^{(N)}_{i_1}\right) ^{b_1}\cdots \left( \eta ^{(N)}_{i_j}\right) ^{b_j} \le \sum _{i=1}^N \left( \eta ^{(N)}_{i}\right) ^{b_1}. \end{aligned}$$

This, together with (6) and (25) yield $\textbf{P}_{\pi ,{{\,\textrm{Coag}\,}}\left( \pi ,\tilde{\pi }\right) } = o\big (c_N\big )$ whenever $b_1\ge 3$. Similarly, whenever $b_2\ge 2$ and using Jensen’s inequality, we have

$$\begin{aligned}&\sum _{\begin{array}{c} i_1,i_2,\dots ,i_j \nonumber \\ \text {all distinct} \end{array}} \left( \eta ^{(N)}_{i_1}\right) ^{b_1}\cdots \left( \eta ^{(N)}_{i_j}\right) ^{b_j} \le \sum _{\begin{array}{c} i_1,i_2\\ \text {distinct} \end{array}} \left( \eta ^{(N)}_{i_1}\right) ^{2}\left( \eta ^{(N)}_{i_2}\right) ^{2}\\&\quad \le \left( \sum _{i=1}^N (\eta ^{(N)}_i)^2\right) ^2 \le \sum _{i=1}^N \eta ^{(N)}_i (\eta ^{(N)}_i)^2. \end{aligned}$$

Thus, (25) now implies $\textbf{P}_{\pi ,{{\,\textrm{Coag}\,}}\left( \pi ,\tilde{\pi }\right) } = o\big (c_N\big )$ whenever $b_2\ge 2$. Since, by hypothesis, we also have $c_N\rightarrow 0$, the convergence to Kingman’s coalescent follows by (ii) of Theorem 2.1. $\square$

## Some examples for neutral evolution

### The Eldon and Wakeley model

Let us consider a simple example of a non-multinomial asymmetric Cannings model, a generalization of the celebrated model studied in Eldon and Wakeley (2006) (see also Huillet and Möhle (2013)), whose genealogy can be easily studied using our criteria.

Let $\left( Y_t^{(N)}\right) _{t\in \mathbb {N}}$ be a collection of i.i.d. r.vs. on [0, 1]. Consider a population where, at any given generation *t*, a randomly chosen individual produces $\lfloor Y_t^{(N)}N\rfloor$ copies of itself which then replace $\lfloor Y_t^{(N)}N\rfloor$ randomly chosen individuals (uniformly and without replacement) in the next generation. Formally, the corresponding random vector of offspring sizes can be constructed as follows. We set $\xi _{K}^{(N)}=\lfloor Y^{(N)}N\rfloor$ for some random variable $Y^{(N)}$ in [0, 1] and some uniformly-distributed index $K\in [N]$. Conditional on those random variables, we set $\xi _{i}^{(N)}=1$ for all $i\in I$, where *I* is a uniform random sample of size $N-\lfloor Y^{(N)}N\rfloor$ from $[N]\setminus \{K\}$ without replacement. Finally we set $\xi _{i}^{(N)}=0$ for all $i\in [N]\setminus \{\{K\}\cup I\}$.

#### Corollary 4.1

Assume that the distribution of $Y^{(N)}$ is given by

$$\begin{aligned} \mathbbm {P}(Y^{(N)}\in dy)=\frac{y^{-2}\Lambda (dy)}{\int _{N^{\frac{\epsilon -1}{2}}}^1 y^{-2}\Lambda (dy)}\mathbbm {1}_{y>N^{\frac{\epsilon -1}{2}}} \end{aligned}$$

for some finite measure $\Lambda$ on [0, 1] and some $\epsilon \in (0,1)$. Then $c_N\sim \mathbbm {E}\left[ \left( Y^{(N)}\right) ^2\right]$ and $\big (\varPi ^{(N,n)}_{\lfloor t/ c_N\rfloor }\big )_{t\ge 0}$ converges weakly in $D([0,\infty ), \mathscr {P}_{n})$ to the $\Lambda$-coalescent.

#### Proof

By the non-heritability condition, the model is equivalent, in terms of its genealogy and in distribution, to the case in which $K{=}1$ almost surely. Furthermore, recalling its definition in (5),

$$\begin{aligned} c_N&= \frac{1}{N(N-1)}\mathbbm {E}[{\lfloor Y^{(N)}N\rfloor (\lfloor Y^{(N)}N\rfloor -1)}] \\&\sim \mathbbm {E}\left[ (Y^{(N)})^2\right] =\frac{\Lambda ([N^{\frac{\epsilon -1}{2}},1])}{\int _{N^{\frac{\epsilon -1}{2}}}^1 y^{-2}\Lambda (dy)}. \end{aligned}$$

Since $\int _{N^{\frac{\epsilon -1}{2}}}^1 y^{-2}\Lambda (dy)\le N^{1-\epsilon }\Lambda ([0,1])$ we have $N c_N\rightarrow \infty$. Then, by Proposition 2.3 we may work with the AWF version of the model with

$$\begin{aligned} \eta _1^{(N)}=\frac{\lfloor Y^{(N)}N\rfloor }{N}= Y^{(N)}+\mathcal {O}\left( N^{-1}\right) =Y^{(N)}(1+\mathcal {O}\left( N^{-1+\frac{1-\epsilon }{2}}\right) ). \end{aligned}$$

Then, by Proposition 3.1, we need only observe that, for all $b\ge 2$,

$$\begin{aligned} \lim _{N\rightarrow \infty } \frac{\mathbbm {E}\left[ (Y^{(N)})^b\right] }{\mathbbm {E}\left[ (Y^{(N)})^2\right] } = \lim _{N\rightarrow \infty } \frac{\int _{N^{\frac{\epsilon -1}{2}}}^1 y^{b-2}\Lambda (dy)}{\int _{N^{\frac{\epsilon -1}{2}}}^1 y^{2-2}\Lambda (dy)} =\frac{\lambda _{b,b}}{ \lambda _{2,2}}. \end{aligned}$$

$$\square $$

### Evolution with recurrent bottlenecks

In Sect. 2.4 we considered a model where the population was left free to increase over *N* and get back to the equilibrium immediately. In this section we model the inverse phenomenon, now the population suddenly and drastically decreases and gets back to the equilibrium immediately after this catastrophic event. Here we adapt our setting to a generalization of the Wright–Fisher model with recurrent bottlenecks introduced in González Casanova et al. (2022). The general picture is the following. At every discrete time, the population undergoes a drastic decrease of its size for only one generation and with a small probability. The next generation then descends from a finite number of individuals that, as $N\rightarrow \infty$, is asymptotically independent of *N*.

More precisely, we consider an AWF model with family frequencies vector

$$\begin{aligned} {\varvec{\eta }}^{(N)}={\varvec{{\bar{\eta }}}}^{(N)}B^{(N)}+{\varvec{{\hat{\eta }}}}^{(N)}(1-B^{(N)}) \end{aligned}$$

where $B^{(N)}$ is a Bernoulli variable that determines whether a bottleneck event occurs. When a bottleneck does occur, the next generation will descend from a random number of individuals $Y^{(N)}<N$ that is independent of $B^{(N)}$, meaning that ${\bar{\eta }}_i^{(N)}=0$ if $i>Y^{(N)}$ (or $\sum _{i=1}^{Y^{(N)}}{\bar{\eta }}_i^{(N)}=1$). We denote by ${\bar{\nu }}_k$ the conditional law of the random probability vector $({\bar{\eta }}_1^{(N)}, \dots , {\bar{\eta }}_k^{(N)})$ given $Y^{(N)}=k$ for $k\ge 1$. We assume that this conditional law does not depend on *N*. Also, we assume that the probability that a bottleneck event occurs is given by

$$\begin{aligned} \mathbbm {P}(B^{(N)}=1)=\frac{\sum _{k=1}^{b_N}F(k)}{{a_N}} \end{aligned}$$

and that, when a bottleneck occurs, there are $k\in [b_N]$ individuals left alive with probability

$$\begin{aligned} \mathbbm {P}(Y^{(N)}=k)=\frac{F(k)}{\sum _{k=1}^{b_N}F(k)}, \end{aligned}$$

where *F* is a (possibly infinite) measure on $\mathbb {N}$. Here $(a_N)_{N\ge 1}$ and $(b_N)_{N\ge 1}$ are two diverging sequences satisfying $\sum _{k=1}^{b_N}F(k)=o(a_n)$ and $b_N=o(N)$. Intuitively, these two assumptions imply that bottlenecks happen with a small probability and that their intensity reduces significantly the population size.

Let us introduce the probability ${\bar{c}}_{N,k}$ that two randomly chosen individuals have the same parent just after a bottleneck of final size *k* occurred, this is given by

$$\begin{aligned} {\bar{c}}_{N,k} =\mathbbm {E}\left[ \sum _{i=1}^k\left( {\bar{\eta }}_i^{(N)}\right) ^2\vert Y^{(N)}=k \right] . \end{aligned}$$

Also consider the same probability when no bottleneck occurred, that is

$$\begin{aligned} {\hat{c}}_{N} =\mathbbm {E}\left[ \sum _{i=1}^N\left( {\hat{\eta }}_{i}^{(N)}\right) ^2\right] . \end{aligned}$$

As will be seen in the next result, two types of genealogies can appear in the limit depending on the frequency of the bottleneck events compared to the rescaling time of the AWF model driven only by ${\varvec{{\hat{\eta }}^{(N)}}}$ (which would correspond to a scenario without bottleneck events). In particular, when bottleneck events dominate the coalescence dynamics, the limit genealogies are described by a $\Xi$-coalescent such that, at rate *F*(*k*), a merging event is performed according to a paintbox driven by the mass partition ${\bar{\nu }}_k$. Denoting by $\varrho _\rho$ the law of a paintbox according to a random mass partition ${\varvec{\rho }}$, in this case the coagulation matrix is given by

26 $$\begin{aligned} {\varvec{\bar{Q}}}^{(n)}(\tilde{\pi }) {:=}\sum _{k=1}^\infty F(k) \int _{\mathscr {P}_{[0,1]}} \varrho _\rho (\tilde{\pi }) {\bar{\nu }}_k(d\rho ). \end{aligned}$$

#### Corollary 4.2

Assume that $0<\lim _{N\rightarrow \infty }\sum _{k=1}^{b_N} F(k){\bar{c}}_{N,k}<\infty$ and that the AWF model driven by ${\varvec{{\hat{\eta }}^{(N)}}}$ is in the domain of attraction of a coalescent with coagulation matrix ${\varvec{Q}}^{(n)}_{\Xi }$, in the sense of Theorem 2.1.

(i) If ${\hat{c}}_Na_N\rightarrow 0$, then, as $N\rightarrow \infty$ the rescaled genealogy $\big (\varPi ^{(N,n)}_{\lfloor ta_N\rfloor }\big )_{t\ge 0}$ converges weakly in the Skorohod space $D([0,\infty ), \mathscr {P}_{n})$ towards a coalescent with coagulation matrix ${\varvec{\bar{Q}}}^{(n)}$ defined in (26) and initial state $\textbf{0}_n$.
(ii) If ${\hat{c}}_Na_N\rightarrow \infty$, then, as $N\rightarrow \infty$ the rescaled genealogy $\big (\varPi ^{(N,n)}_{\lfloor t/{\hat{c}}_N\rfloor }\big )_{t\ge 0}$ converges weakly in the Skorohod space $D([0,\infty ), \mathscr {P}_{n})$ towards a coalescent with coagulation matrix ${\varvec{Q}}^{(n)}_{\Xi }$ and initial state $\textbf{0}_n$.
(iii) If ${\hat{c}}_Na_N\rightarrow \ell >0$, then, as $N\rightarrow \infty$ the rescaled genealogy $\big (\varPi ^{(N,n)}_{\lfloor ta_N\rfloor }\big )_{t\ge 0}$ converges weakly in the Skorohod space $D([0,\infty ), \mathscr {P}_{n})$ towards a coalescent with coagulation matrix ${\varvec{\bar{Q}}}^{(n)}_{\Xi }+ \ell {\varvec{Q}}^{(n)}_{\Xi }$ and initial state $\textbf{0}_n$.

#### Proof

Conditioning on the value of $B^{(N)}$, we obtain that the coagulation probabilities ${\varvec{P}}^{(N,n)}$ are given by

$$\begin{aligned} {\varvec{P}}^{(N,n)}(\tilde{\pi })&= \frac{\sum _{k=1}^{b_N}F(k)}{a_N} \sum _{k=1}^{b_N} \frac{ F(k)}{\sum _{k=1}^{b_N}F(k)} \int _{\mathscr {P}_{[0,1]}} \varrho _\rho (\tilde{\pi }) {\bar{\nu }}_k(d\rho ) \\&\quad + \left( 1-\frac{\sum _{k=1}^{b_N}F(k)}{a_N}\right) \int _{\mathscr {P}_{[0,1]}} \varrho _\rho (\tilde{\pi }) {\hat{\nu _N}}(d\rho ). \end{aligned}$$

where ${\hat{\nu _N}}$ is law of ${\varvec{{\hat{\eta }}^{(N)}}}$. In particular, computing $c_N$ (in (2)), we have

$$\begin{aligned} c_N&=\frac{\sum _{k=1}^{b_N}F(k){\bar{c}}_{N,k}}{a_N}+(1-\frac{\sum _{k=1}^{b_N}F(k)}{a_N}){\hat{c}}_N. \end{aligned}$$

In case (i), we get that

$$\begin{aligned} c_N\sim \frac{\sum _{k=1}^{b_N}F(k){\bar{c}}_{N,k}}{a_N} \end{aligned}$$

and since $0<\lim _{N\rightarrow \infty }\sum _{k=1}^{b_N} F(k){\bar{c}}_{N,k}<\infty$, the limit coagulation rate matrix obtained, after rescaling time by $1/a_N$ instead of $c_N$, consists in the paintbox representation obtained in (26).

In case (ii), we get that

$$\begin{aligned} c_N\sim {\hat{c}}_N \end{aligned}$$

and we are coming back to the AWF model driven by ${\varvec{\hat{\eta }}}^{(N)}$, with limit coagulation matrix ${\varvec{Q}}^{(n)}_{\Xi }$.

Finally, in case (iii), we get that

$$\begin{aligned} c_N\sim \frac{\sum _{k=1}^{b_N}F(k){\bar{c}}_{N,k}}{a_N}+\frac{\ell }{a_N} \end{aligned}$$

so that after rescaling time by $1/a_N$ the effects of both coagulation matrices add up in the limit. $\square$

We provide two examples.

- Wright–Fisher model with Wright–Fisher bottlenecks: The case where ${\varvec{\bar{\eta }}}^{(N)}=(\frac{1}{Y^{(N)}},\dots ,\frac{1}{Y^{(N)}},0,\dots ,0)$, ${\varvec{\hat{\eta }}}^{(N)}=(\frac{1}{N},\dots ,\frac{1}{N})$, and also $a_N=N^\alpha$ for some $\alpha \in (0,1)$ and *F* is any measure on $\mathbb {N}$ such that $\sum F(k)/k<\infty$, is similar to the Wright–Fisher model with short drastic recurrent bottlenecks studied in González Casanova et al. (2022). The limit genealogies are described by the so-called symmetric coalescent. Its coagulation matrix is given by (26) where ${\bar{\nu }}_k=\delta _{(\frac{1}{k},\dots ,\frac{1}{k})}$.
- Wright–Fisher model with general Dirichlet bottlenecks: A normalized random partition with *K* components is a partition of the form $(\frac{W_1}{S_K},\dots , \frac{W_K}{S_K})$ where $(W_1, W_2,\dots )$ is a family of i.i.d. random variables with finite variance and $S_{K}=\sum _{i=1}^KW_i$. The model in which ${\varvec{\bar{\eta }}}^{(N)}$ is a normalized random partition with $Y^{(N)}$ components, ${\varvec{\hat{\eta }}}^{(N)}=(\frac{1}{N},\dots ,\frac{1}{N})$, and also $a_N=N^\alpha$ for some $\alpha \in (0,1)$ and *F* is any measure on $\mathbb {N}$ such that $\sum F(k)/k<\infty$, provides an illustrative example where the limit genealogies are described by the Dirichlet coalescents introduced in González Casanova et al. (2024).

## An example for selective evolution

In this section we study the case in which the family frequencies of the AWF model are constructed from a size-biased pick $({\tilde{V}}_1, {\tilde{V}}_2,\dots )$ of a Poisson–Dirichlet (PD) random mass partition of parameters $(\alpha ,\theta )$, $0<\alpha <1, \theta >-\alpha$ (Pitman and Yor 1997), by setting, for $1\le i \le N$,

27 $$\begin{aligned} \eta ^{(N)}_i = \frac{{\tilde{V}}_{i}^\gamma }{\sum _{j=1}^N {\tilde{V}}_j^\gamma }, \end{aligned}$$

where $\gamma \in \mathbb {R}$ is a parameter of the model.

The case $\gamma =\alpha$ of the following theorem was proved in Cortines and Mallein (2017). Here we extend their results by following their main heuristics but using different technical arguments such as applying Propositions 3.1 and 3.2 instead of their Lemmas 3.1 and 3.2, thus avoiding having to deal with the largest family frequency among ${\varvec{\eta }}^{(N)}$ directly. To ease notations we write

$$\begin{aligned} u_N=\sum _{i=1}^N i^{-\gamma /\alpha } \end{aligned}$$

in the whole section.

### Theorem 5.1

Assume $\alpha /2<\gamma \le \alpha$ and set $L_N = \ell _{\alpha ,\theta ,\gamma }u_N^{1+\frac{\theta }{\alpha }}$ where

$$\begin{aligned} \ell _{\alpha ,\theta ,\gamma }^{-1}= \frac{\alpha }{\gamma }\frac{\Gamma (1-\alpha )^{\frac{\theta }{\alpha }}}{\Gamma (1+\gamma -\alpha )^{1+\frac{\theta }{\alpha }}} \frac{\Gamma \left( \frac{\alpha +\theta }{\alpha }(1-\frac{\gamma }{\alpha })+1\right) }{\Gamma \left( \left( \alpha +\theta \right) \left( 1-\frac{\gamma }{\alpha }\right) +1\right) } \frac{\Gamma (1+\theta ) \Gamma \left( 1-\frac{\theta }{\alpha }\right) }{\textsf {B}\left( 1-\frac{\theta }{\alpha },1+\frac{\theta }{\alpha }\right) }. \end{aligned}$$

Let $\big (\varPi ^{(N,n)}_{\lfloor t/c_N\rfloor }\big )_{t\ge 0}$ be the genealogy of the AWF model with family frequencies as in (27).

(i) If $\theta \in (-\alpha ,\alpha )$, then $c_N\sim \left( 1-\frac{\theta }{\alpha }\right) /L_N$ and $\big (\varPi ^{(N,n)}_{\lfloor t/c_N\rfloor }\big )_{t\ge 0}$ converges weakly in the Skorohod topology for $D([0,\infty ), \mathscr {P}_{n})$ to the Beta$\left( 1-\frac{\theta }{\alpha },1+\frac{\theta }{\alpha }\right)$-coalescent, as $N\rightarrow \infty$.
(ii) If $\theta =\alpha$, then $c_N=\mathcal {O}\left( u_N^{-2}\log \left( u_N\right) \right)$, whereas if $\theta >\alpha$, then $c_N=\mathcal {O}\left( u_N^{-2}\right)$. In both cases $\big (\varPi ^{(N,n)}_{\lfloor t/c_N\rfloor }\big )_{t\ge 0}$ converges weakly to Kingman’s coalescent, as $N\rightarrow \infty$.

Interestingly, the choice of $\gamma$ does not affect the shape of the limit genealogy, but only the time rescaling. As explained in the following section, the above Theorem 5.1 will provide a characterization of the limit genealogy of a population model that incorporates noisy natural selection (see the upcoming Theorem 5.3).

### Motivation: exponential models

Here we briefly explain how Theorem 5.1 describes the limit genealogy of a slightly more general exponential model (EM) than those studied in Cortines and Mallein (2017), Cortines and Mallein (2018), which is in fact a simple combination of them both. In order to avoid plain repetitions of their arguments we will heavily refer to their results and proofs, and only provide the main heuristics therein.

We now describe the $(N,\beta ,\kappa )$-EM, a generalization of the $(N,\beta )$-EM described in Cortines and Mallein (2017). Consider a population of particles positioned on the real line that evolve through discrete generations $t\in \mathbb {N}$. Every generation is of size *N* and is constructed from the previous generation through branching and selection steps. The positions of the particles, say $(X_1^{(N)}(t),\dots ,X_N^{(N)}(t))$, give individuals’ fitness levels: higher position gives a greater probability of having more descendants in the next generation. Let $\kappa >0$ be a parameter of the model. During the branching step an individual at position *x* will be replaced, independently from the other particles and from the previous generations, by a countable number of children whose positions are given by a PPP of intensity $e^{-(s-\kappa x)}\hspace{3pt}ds$. Let $\beta >1$ be another parameter of the model. During the selection step, *N* of the newly produced particles are sampled without replacement, and with the probability of picking a child at position *x* being proportional to $e^{\beta x}$. Proposition 1.3 in Cortines and Mallein (2017) ensures that the model is well defined (i.e. the selection probabilities are well defined) precisely when $\beta >1$. The case $\kappa =1$ corresponds to the $(N,\beta )$-EM in which children particles are centered around the position of the parent, as opposed to the general case in which children are positioned around the scaled position of the parent $\kappa x$. On the other hand, if instead of the noisy selection step just described we performed a “perfect” selection step, i.e. if we selected the *N* rightmost children (which corresponds to setting $\beta =\infty$), then we recover the exactly solvable model studied in Cortines and Mallein (2018). Another variation of the exponential model is also studied by Schertzer and Wences (2023), in which the selection step is performed on the subset of the $\lfloor N^\chi \rfloor$, $\chi \ge 1$, rightmost children produced during the branching step. In this case the strength of selection $\beta$ may range in $[0,\infty )$, finding both discrete and continuous time coalescent processes in the limit genealogy, depending on the choice of $\chi$ and $\beta$.

In the following proposition we recapitulate without formal proofs some of the results in Cortines and Mallein (2017) which will establish a clear connection between the genealogy of the $(N,\beta ,\kappa )$-EM and the AWF model of Theorem 5.1.

#### Proposition 5.2

In the $(N,\beta , \kappa )$-EM, a particle produced during the branching step of generation *t* is a child of the particle at position $X_i^{(N)}(t)$ with probability, conditional on $(X_1^{(N)}(t),\dots ,X_N^{(N)}(t))$ and independently from its own position and from the rest of its siblings, given by $\frac{e^{\kappa X^{(N)}_i(t)}}{\sum _{j=1}^N e^{\kappa X^{(N)}_j(t)}}.$ Furthermore, the sequence of random vectors $\left( \frac{e^{\kappa X^{(N)}_1(t)}}{\sum _{j=1}^N e^{\kappa X^{(N)}_j(t)}}, \dots , \frac{e^{\kappa X^{(N)}_N(t)}}{\sum _{j=1}^N e^{\kappa X^{(N)}_j(t)}}\right) _{t\in \mathbb {N}}$ is independent and identically distributed as $\left( \frac{{\tilde{V}}_{1}^{\kappa /\beta }}{\sum _{j=1}^N {\tilde{V}}_j^{\kappa /\beta } },\dots ,\frac{{\tilde{V}}_{N}^{\kappa /\beta }}{\sum _{j=1}^N {\tilde{V}}_j^{\kappa /\beta } } \right)$ where $({\tilde{V}}_1,{\tilde{V}}_2, \cdots )$ is a size-biased pick of a PD$(1/\beta ,0)$ random variable.

#### Sketch of the proof

This follows from Lemma 1.6 and Proposition 4.1 in Cortines and Mallein (2017); see also the proof of Theorem 1.2 therein. Here we only present the main heuristics. To ease the notation, let us set $\kappa =1$.

The first assertion follows from well-known identities of PPPs. Indeed, by superimposing *N* independent PPPs of respective intensities $e^{-(s-X^{(N)}_i(t))}ds, 1\le i\le N,$ one can produce a single (joint) PPP of intensity $e^{-(s-X^{(N)}_{eq}(t))}ds$ where $X^{(N)}_{eq}(t)=\log \left( \sum _{i=1}^N e^{X^{(N)}_i(t)}\right)$. Then, a particle at position *x* of the joint PPP happens to be a particle of the *i*th PPP, i.e. is a daughter of the particle at position $X^{(N)}_i(t)$, with probability

$$\begin{aligned} \frac{e^{-(x-X^{(N)}_i(t))}}{\sum _{j=1}^N e^{-(x-X^{(N)}_j(t))}}= \frac{e^{X^{(N)}_i(t)}}{\sum _{j=1}^N e^{X^{(N)}_j(t)}}. \end{aligned}$$

The i.i.d. nature of the sequence $\left( \frac{e^{X^{(N)}_1(t)}}{\sum _{j=1}^N e^{X^{(N)}_j(t)}}, \dots , \frac{e^{X^{(N)}_N(t)}}{\sum _{j=1}^N e^{X^{(N)}_j(t)}}\right) _{t\in \mathbb {N}}$ follows from observing that the positions of the children produced during the branching step of generation *t* are distributed like $X^{(N)}_{eq}(t)+z_i, i\ge 1$ where $(z_1,z_2,\dots )$ are the atoms of a PPP of intensity $e^{-s}ds$, arranged in decreasing order, and this point process is independent of all the previous generations. Hence, we can write the sampling probabilities of the respective selection step (to produce generation $t+1$) as

$$\begin{aligned} \left( \frac{e^{\beta X^{(N)}_{eq}(t) + \beta z_i}}{\sum _{{j\ge 1}} e^{\beta X^{(N)}_{eq}(t) + \beta z_{j}}} \right) _{i\in \mathbb {N}} =\left( \frac{e^{\beta z_i}}{\sum _{{j\ge 1}} e^{ \beta z_{j}}} \right) _{i\in \mathbb {N}}. \end{aligned}$$

Observe that the latter does not depend on $(X^{(N)}_1(t),\dots ,X^{(N)}_N(t))$ and is PD($1/\beta ,0$)-distributed, according to the mapping theorem (Kingman 1992) and Proposition 10 in Pitman and Yor (1997). Furthermore, if $\left( Z_1,\dots , Z_N\right)$ is a sample without replacement from $(z_1,z_2, \dots )$ with probabilities given by the r.h.s. above, then we can write the family frequencies of generation $t+1$ as, for $1\le i\le N$,

$$\begin{aligned} \frac{e^{X^{(N)}_i(t+1)}}{\sum _{j=1}^N e^{X^{(N)}_j(t+1)}} \overset{d}{=}\frac{e^{X^{(N)}_{eq}(t)+Z_i}}{\sum _{j=1}^N e^{X^{(N)}_{eq}(t)+Z_j}} =\frac{e^{Z_i}}{\sum _{j=1}^N e^{Z_j}} \overset{d}{=}\frac{{\tilde{V}}_{i}^{1/\beta }}{\sum _{j=1}^N {\tilde{V}}_j^{1/\beta } }. \end{aligned}$$

The r.h.s. above is independent of both $(X^{(N)}_1(t),\dots ,X^{(N)}_N(t))$ and *t*, and thus i.i.d over *t*. $\square$

As a straightforward consequence of point (i) of Theorem 5.1 with $\alpha =1/\beta$, $\theta =0$, and $\gamma =\kappa / \beta$, we obtain the following result.

#### Theorem 5.3

Consider a $(N,\beta ,\kappa )$-EM with $\beta >1$ and $\kappa \in (1/2,1]$. Then, as $N\rightarrow \infty$, $c_N\sum _{i=1}^N i^{-\kappa }\rightarrow \frac{\Gamma \left( 2-\kappa \right) }{\kappa \Gamma \left( 1-\frac{1-\kappa }{\beta }\right) \Gamma \left( 1+\frac{1+\kappa }{\beta }\right) }$ and the rescaled genealogical process $\big (\varPi ^{(N,n)}_{\lfloor t/c_N\rfloor }\big )_{t\ge 0}$ converges weakly in the Skorohod topology for $D([0,\infty ), \mathscr {P}_{n})$ to the Bolthausen–Sznitman coalescent.

Comparing this result with Theorem 1.1 in Cortines and Mallein (2017) we see that even in the range $\kappa \in (1/2,1)$, and in contrast to their model in which other Beta-coalescents appear in the limit, in our case the limit genealogy is always described by the Bolthausen-Sznitman coalescent. Also, in contrast to many other examples in the literature, our convergence to the Bolthausen-Sznitman coalescent may occur at a time scale other than logarithmic on *N*; whenever $\kappa \in (1/2,1)$ we have $c_N=\mathcal {O}\left( \sum _{i=1}^N i^{-\kappa }\right) =\mathcal {O}\left( N^{1-\kappa }\right)$.

### Martingales associated to the Poisson–Dirichlet distribution

Before proving Theorem 5.1 we develop some general results on the PD($\alpha ,\theta$) distribution. We provide an understanding of the asymptotic behavior of the normalizing sum appearing in (27), mainly

$$\begin{aligned} \zeta _{N,\gamma }{:=}\sum _{i=1}^N {\tilde{V}}_i^\gamma . \end{aligned}$$

For this we recall the well-known stick-breaking construction of PD$(\alpha ,\theta )$ size-biased picks. The sequence $({\tilde{V}}_1,{\tilde{V}}_2,\dots )$ may be constructed from a collection $(Y_1, Y_2, \dots )$ of independent random variables where $Y_i$ is Beta($1-\alpha , \theta + i\alpha$) distributed, by setting

$$\begin{aligned} {\tilde{V}}_1 = Y_1, \text { and }{\tilde{V}}_i = (1-Y_1)\dots (1-Y_{i-1})Y_i. \end{aligned}$$

This can be found in Proposition 2 of Pitman and Yor (1997).

We now write $\zeta _{N,\gamma }$ in terms of the sequence $(Y_1, Y_2, \dots )$. For this observe that

28 $$\begin{aligned} \prod _{i=1}^{N}(1-Y_i)^\gamma = e^{-\gamma \mu _N}e^{\gamma S_N} \end{aligned}$$

where $S_N= \mu _N+\sum _{i=1}^N \log (1-Y_i)$ defines a martingale when $\mu _N= \sum _{i=1}^N -\mathbbm {E}\left[ \log (1-Y_i)\right]$. We also define the martingale

$$\begin{aligned} M_{N,\gamma }&{:=}\sum _{i=1}^N \left( i^{\gamma -1} Y_i^\gamma - \mathbbm {E}\left[ i^{\gamma -1}Y_i^{\gamma }\right] \right) . \end{aligned}$$

With this, we have

29 $$\begin{aligned} \zeta _{N,\gamma }&= \sum _{i=1}^N Y_i^\gamma e^{-\gamma \mu _{i-1}}e^{\gamma S_{i-1}}\nonumber \\&= \sum _{i=1}^N \left( M_{i,\gamma } - M_{i-1,\gamma } + \mathbbm {E}\left[ i^{\gamma -1}Y_i^{\gamma }\right] \right) i^{1-\gamma }e^{-\gamma \mu _{i-1}} e^{\gamma S_{i-1}}\nonumber \\&= \overline{M}_{N,\gamma } + \Sigma _{N,\gamma } \end{aligned}$$

where $\overline{M}_{N,\gamma }$ is the martingale

$$\begin{aligned} \overline{M}_{N,\gamma } {:=}\sum _{i=1}^N (M_{i,\gamma } - M_{i-1,\gamma }) i^{1-\gamma }e^{-\gamma \mu _{i-1}}e^{\gamma S_{i-1}} \end{aligned}$$

and $\Sigma _{N,\gamma }$ is the sum

$$\begin{aligned} \Sigma _{N,\gamma }{:=}\sum _{i=1}^N \frac{\textsf {B}(1+\gamma -\alpha ,\theta +i\alpha )}{\textsf {B}(1-\alpha ,\theta +i\alpha )}e^{-\gamma \mu _{i-1}}e^{\gamma S_{i-1}} \end{aligned}$$

since $$\mathbbm {E}\left[ Y_i^{\gamma }\right] ={\textsf {B}(1+\gamma -\alpha ,\theta +i\alpha )}/{\textsf {B}(1-\alpha ,\theta +i\alpha )}$$.

We now study the product $\prod _{i=1}^N (1-Y_i)^\gamma$ with the help of the martingale $S_N$ and equation (28). The following lemma is an adaptation of Lemma 2.4 of Cortines and Mallein (2017) into our setting. Let $\psi (z)=\frac{d}{dz}\log \Gamma (z)$ be the digamma function, and for $a>-1$ let $\Upupsilon _a$ be the constant such that $-\log (N) + \sum _{i=1}^N (a + i)^{-1}=\Upupsilon _a + o\big (1\big )$, e.g. $\Upupsilon \equiv \Upupsilon _0$ is the Euler-Mascheroni constant.

#### Lemma 5.4

There exists a random variable $S_\infty$ such that

$$\begin{aligned} \lim _{N\rightarrow \infty } e^{\gamma S_N} = e^{\gamma S_\infty } \end{aligned}$$

almost surely. Furthermore, letting $K_{\alpha ,\theta }=\exp \{\psi (\theta +1)-\frac{1}{\alpha }\Upupsilon _{\theta /\alpha }\}$, the convergence also holds in $\textsf {L}^{1}$ whenever $\gamma > -(\theta + \alpha )$ and, in this case,

$$\begin{aligned} \mathbbm {E}\left[ e^{\gamma S_\infty }\right] = K_{\alpha ,\theta }^\gamma \frac{\Gamma (\theta +1)}{\Gamma (\theta +\gamma +1)} \frac{\Gamma \left( \frac{\theta +\gamma }{\alpha }+1\right) }{\Gamma \left( \frac{\theta }{\alpha }+1\right) }. \end{aligned}$$

#### Proof

First recall that if *X* is Beta(*a*, *b*) distributed, then $\mathbbm {E}\left[ \log (X)\right] = \psi (a)-\psi (a+b).$ So we have $-\mathbbm {E}\left[ \log (1-Y_i)\right] =\psi (\theta + (i-1)\alpha + 1)-\psi (\theta + i\alpha )$. Using the well-known identity $\psi (z+1)=\psi (z)+z^{-1}$, the bounds $\log (z)-z^{-1}\le \psi (z) \le \log (z+1)-z^{-1}$ for $z>0$ (see Corollary 2.3 in Muqattash and Yahdi (2006)), and the definition of $\Upupsilon _{\theta /\alpha }$, we obtain, as $N\rightarrow \infty$,

30 $$\begin{aligned} \mu _N&= \psi (\theta +1)-\psi (\theta +N\alpha )+\sum _{i=1}^{N-1} \frac{1}{\theta + i\alpha }\nonumber \\&= \psi (\theta +1) - \log (N) - \log (\alpha ) + \frac{1}{\alpha }\log (N) - \frac{1}{\alpha }\Upupsilon _{\theta /\alpha } + o\big (1\big ). \end{aligned}$$

Thus, we arrive at

31 $$\begin{aligned} e^{\gamma \mu _N} \sim \alpha ^{-\gamma } K_{\alpha ,\theta }^\gamma N^{\gamma (1-\alpha )/\alpha } \end{aligned}$$

for any $\gamma \in \mathbb {R}$. This, together with (28) and Lemma 2.4 in Cortines and Mallein (2017), implies the stated a.s. and $\textsf {L}^{p}$ convergences for $e^{\gamma S_N}$ to some strictly positive random variable, so that $S_N$ itself converges to a (finite) r.v. $S_\infty$ (in fact one can also check that $S_N$ is itself an $\textsf {L}^{2}$-bounded martingale, we avoid such computations here). $\square$

We now study the asymptotic behavior of the two terms, $\overline{M}_{N,\gamma }$ and $\Sigma _{N,\gamma }$, that compose $\zeta _{N,\gamma }$ in (29).

#### Lemma 5.5

Assume $\gamma > \alpha /2$, then there exists a r.v. $\overline{M}_{\infty ,\gamma }$ such that

$$\begin{aligned} \lim _{N\rightarrow \infty } \overline{M}_{N,\gamma } = \overline{M}_{\infty ,\gamma } \end{aligned}$$

almost surely and in $\textsf {L}^{2}$. As a straightforward consequence, if $\gamma \le \alpha$, then

$$\begin{aligned} \lim _{N\rightarrow \infty } \frac{\overline{M}_{N,\gamma }}{u_N} = 0 \end{aligned}$$

almost surely and in $\textsf {L}^{2}$.

#### Proof

We compute, for every $\gamma >0$,

$$\begin{aligned} \mathbbm {E}\left[ (M_{i,\gamma }-M_{i-1,\gamma })^2\right]&=\mathbbm {Var}\left( i^{\gamma -1}Y_i^\gamma \right) \\ &= \frac{i^{-2}}{\alpha ^{2\gamma }}\left( \frac{\Gamma (1+2\gamma -\alpha )}{\Gamma (1-\alpha )}-\frac{\Gamma (1+\gamma -\alpha )^2}{\Gamma (1-\alpha )^2} + \mathcal {O}\left( i^{-1}\right) \right) \end{aligned}$$

as $i\rightarrow \infty$ so that, using (31), Lemma 5.4, and the condition $\gamma >{\alpha }/{2}$

$$\begin{aligned}&\sum _{i=1}^\infty \mathbbm {Var}\left( \overline{M}_{i+1,\gamma }-\overline{M_{i,\gamma }}\right) \\&=\sum _{i=1}^\infty i^{-2{\gamma }/{\alpha }} \mathbbm {E}\left[ e^{2\gamma S_{i}}\right] \alpha ^{-2\gamma }\left( \frac{\Gamma (1+2\gamma -\alpha )}{\Gamma (1-\alpha )}-\frac{\Gamma (1+\gamma -\alpha )^2}{\Gamma (1-\alpha )^2}\right) (1+\mathcal {O}\left( i^{-1}\right) )\\&\le C\mathbbm {E}\left[ e^{2\gamma S_{\infty }}\right] \sum _{i=1}^\infty i^{-2\gamma /\alpha } <\infty \end{aligned}$$

for some $C>0$. This implies the a.s. and $\textsf {L}^{2}$ convergence of $\overline{M}_{N,\gamma }$ to some r.v. $\overline{M}_{\infty ,\gamma }$. $\square$

#### Lemma 5.6

We have, for $\gamma \le \alpha$,

$$\begin{aligned} \lim _{N\rightarrow \infty } \frac{\Sigma _{N,\gamma }}{u_N} = \frac{\Gamma (1+\gamma -\alpha )}{\Gamma (1-\alpha )} K_{\alpha ,\theta }^{-\gamma } e^{\gamma S_\infty } \end{aligned}$$

almost surely and in $\textsf {L}^{1}$.

#### Proof

Observe that, as $N\rightarrow \infty$,

32 $$\begin{aligned} \frac{\textsf {B}(1+\gamma -\alpha ,\theta +N\alpha )}{\textsf {B}(1-\alpha ,\theta +N\alpha )} = \frac{\alpha ^{-\gamma }\Gamma (1+\gamma -\alpha )}{\Gamma (1-\alpha )}N^{-\gamma }\left( 1+\mathcal {O}\left( N^{-1}\right) \right) . \end{aligned}$$

Then equation (31) together with Lemma 5.4 and the Stolz-Césaro theorem ($u_N$ is monotone and diverging) in the first equality below, yield

$$\begin{aligned} \lim _{N\rightarrow \infty } \frac{\Sigma _{N,\gamma }}{u_N}&{=\lim _{N\rightarrow \infty } \frac{\Sigma _{N,\gamma }-\Sigma _{N-1,\gamma }}{u_N-u_{N-1}}}\\&{=}\frac{\alpha ^{-\gamma }\Gamma (1+\gamma -\alpha )}{\Gamma (1-\alpha )}\lim _{N\rightarrow \infty } \frac{ N^{-\gamma }e^{-\gamma \mu _{N-1}}e^{\gamma S_{N-1}}}{N^{-{\gamma }/{\alpha }}}\\&{=}\frac{\alpha ^{-\gamma }\Gamma (1+\gamma -\alpha )}{\Gamma (1-\alpha )} \alpha ^\gamma K_{\alpha ,\theta }^{-\gamma } e^{\gamma S_\infty } \end{aligned}$$

almost surely. Hence the desired a.s. convergence follows. Similarly, using again the Stolz-Césaro theorem, by the same equation (31) and Lemma 5.4,

$$\begin{aligned} \lim _{N\rightarrow \infty } \frac{ \mathbbm {E}\left[ \Sigma _{N,\gamma }\right] }{u_N} =\frac{\Gamma (1+\gamma -\alpha )}{\Gamma (1-\alpha )} K_{\alpha ,\theta }^{-\gamma } \mathbbm {E}\left[ e^{\gamma S_\infty }\right] \end{aligned}$$

so that Scheffe’s lemma gives the corresponding $\textsf {L}^{1}$ convergence. $\square$

Thanks to the previous lemmas, we obtain the asymptotic behavior of $\zeta _{N,\gamma }$.

#### Proposition 5.7

If $$\alpha /2< \gamma \le \alpha $$, then

$$\begin{aligned} \lim _{N\rightarrow \infty } \frac{\zeta _{N,\gamma }}{u_N} = \frac{\Gamma (1+\gamma -\alpha )}{\Gamma (1-\alpha )} K_{\alpha ,\theta }^{-\gamma } e^{\gamma S_\infty } \end{aligned}$$

almost surely and in $\textsf {L}^{1}$. Moreover, if $\eta \gamma <\theta +\alpha$, then

33 $$\begin{aligned} \lim _{N\rightarrow \infty } \left( \frac{\zeta _{N,\gamma }}{u_N}\right) ^{-\eta } = \left( \frac{\Gamma (1+\gamma -\alpha )}{\Gamma (1-\alpha )}K_{\alpha ,\theta }^{-\gamma } e^{\gamma S_\infty }\right) ^{-\eta } \end{aligned}$$

almost surely and in $\textsf {L}^{1}$.

On the other hand, if $\gamma >\alpha$, then there exists a r.v. $\zeta _{\infty ,\gamma }$ such that

34 $$\begin{aligned} \lim _{N\rightarrow \infty } \zeta _{N,\gamma } = \zeta _{\infty ,\gamma } \end{aligned}$$

almost surely and in $\textsf {L}^{p}$ for $p\ge 1$.

#### Proof

We first assume $\gamma \le \alpha$. The a.s. and $\textsf {L}^{1}$ convergences in this case follow directly from (29) and Lemmas 5.5 and 5.6. The second convergences will follow once we prove

35 $$\begin{aligned} \limsup _{N\ge 0}\mathbbm {E}\left[ \left( \frac{u_N}{\zeta _{N,\gamma }}\right) ^{\eta }\right] <\infty \end{aligned}$$

together with an application of dominated convergence and Scheffe’s lemma. To upperbound the expectations in (35) define the discrete probability space $\Omega =\{f(1),\dots ,f(N)\}$ where $f(i){:=}Y_i^\gamma e^{-\gamma \mu _{i-1}}e^{\gamma S_{i-1}}i^{{\gamma }/{\alpha }}$ with probability measure

$$\begin{aligned} \forall A\subset \Omega ,\quad \mathbbm {P}_\Omega \left( A\right) =\frac{1}{u_N}\sum _{i=1}^N i^{-{\gamma }/{\alpha }}\mathbbm {1}_{f(i)\in A}. \end{aligned}$$

Then, by Jensen’s inequality we have

$$\begin{aligned} {\zeta _{N,\gamma }^{-\eta }}&=\exp \left\{ -\eta \log \left( \sum _{i=1}^N Y_i^\gamma e^{-\gamma \mu _{i-1}}e^{\gamma S_{i-1}}i^{{\gamma }/{\alpha }}i^{-{\gamma }/{\alpha }}\right) \right\} \\&=e^{-\eta \log \left( u_N\right) } e^{ -\eta \log \left( \mathbbm {E}_\Omega \left[ x\right] \right) } \\&\le e^{-\eta \log \left( u_N\right) } e^{- \eta \mathbbm {E}_\Omega \left[ \log (x)\right] }\\&= \left( u_N\right) ^{-\eta } \exp \left\{ - \frac{\eta \gamma }{u_N}\sum _{i=1}^N \frac{\log (Y_i)-\mu _{i-1}+S_{i-1}+\frac{1}{\alpha }\log (i)}{i^{{\gamma }/{\alpha }}}\right\} . \end{aligned}$$

Furthermore, by Lemma 5.4 and the condition $\eta \gamma <\alpha +\theta$ we have, for $\epsilon >0$ small enough and using that $\left( e^{-\eta \gamma (1+\epsilon )S_N}\right) _{N\le 1}$ is a submartingale,

$$\begin{aligned} \exp \left\{ -\eta \gamma (1+\epsilon ) \frac{\sum _{i=1}^N S_{i-1}i^{-{\gamma }/{\alpha }}}{u_N} \right\} \le \sup _{N\in \mathbb {N}} e^{-\eta \gamma (1+\epsilon ) S_{N}}\in \textsf {L}^{1}. \end{aligned}$$

Thus, plugging these estimates and using Hölder’s inequality ($p=1+\epsilon$),

36 $$\begin{aligned}&\mathbbm {E}\left[ \left( \frac{u_N}{\zeta _{N,\gamma }}\right) ^{\eta }\right] \nonumber \\&\le \left\Vert \sup _{N\in \mathbb {N}} e^{-\eta \gamma (1+\epsilon ) S_{N}} \right\Vert _{{1}} \mathbbm {E}\left[ \exp \left\{ -\frac{1+\epsilon }{\epsilon } \frac{\eta \gamma }{u_N} \sum _{i=1}^N \frac{\log (Y_i)-\mu _{i-1}+\frac{1}{\alpha }\log (i)}{i^{{\gamma }/{\alpha }}} \right\} \right] ^{\frac{\epsilon }{1+\epsilon }}. \end{aligned}$$

We now compute, for *N* large enough to ensure $\frac{1+\epsilon }{\epsilon } \frac{\eta \gamma }{u_N}<1-\alpha$, and using an easy consequence of the mean value theorem on the function $z\rightarrow \log \Gamma (z)$,

$$\begin{aligned}&\mathbbm {E}\left[ \exp \left\{ -\eta \gamma \frac{1+\epsilon }{\epsilon } \frac{\log (Y_i)i^{-{\gamma }/{\alpha }}}{u_N} \right\} \right] =\mathbbm {E}\left[ Y_i^{-\eta \gamma \frac{1+\epsilon }{\epsilon } \frac{i^{-{\gamma }/{\alpha }}}{u_N}}\right] \\&= \exp \left\{ \log \Gamma \left( 1-\alpha -\eta \gamma \frac{1+\epsilon }{\epsilon }\frac{i^{-{\gamma }/{\alpha }}}{u_N}\right) -\log \Gamma \left( 1-\alpha \right) \right\} \\&\times \exp \left\{ \log \Gamma (1+\theta +(i-1)\alpha )- \log \Gamma \left( 1+\theta +(i-1)\alpha - \eta \gamma \frac{1+\epsilon }{\epsilon }\frac{i^{-{\gamma }/{\alpha }}}{u_N}\right) \right\} \\&\le \exp \left\{ \eta \gamma \frac{1+\epsilon }{\epsilon }\frac{i^{-{\gamma }/{\alpha }}}{u_N}\psi \left( 1+\theta +(i-1)\alpha \right) \right\} . \end{aligned}$$

Equation (30) and the identity $\psi (1+\theta + (i-1)\alpha )=\psi (\theta + (i-1)\alpha ) + \frac{1}{\theta + (i-1)\alpha }$ yield, as $i\rightarrow \infty$,

$$\begin{aligned} \psi \left( 1+\theta +(i-1)\alpha \right) -\mu _{i-1}+\frac{1}{\alpha }\log (i) =\frac{1}{\theta + (i-1)\alpha } -\psi (\theta +1) + \frac{1}{\alpha }\Upupsilon _{\theta /\alpha } + o\big (1\big ). \end{aligned}$$

Thus we obtain, for all $i\le N$, that there exists $C'>0$ such that

$$\begin{aligned} \mathbbm {E}\left[ \exp \left\{ -\frac{\eta \gamma }{u_N}\frac{1+\epsilon }{\epsilon } {i^{-{\gamma }/{\alpha }}(\log (Y_i)-\mu _{i-1}+\frac{1}{\alpha }\log (i))} \right\} \right] ^{\frac{\epsilon }{1+\epsilon }} \le \exp \left\{ C'\frac{\eta \gamma }{u_N} {i^{-{\gamma }/{\alpha }}} \right\} . \end{aligned}$$

Taking the product over *i*, and plugging in (36), we conclude, for all $N\in \mathbb {N}$,

$$\begin{aligned} \mathbbm {E}\left[ \left( \frac{u_N}{\sum _{i=1}^N {\tilde{V}}_i^\gamma }\right) ^{\eta }\right] <\exp \{C'\eta \gamma \}, \end{aligned}$$

which entails (35).

We now prove (34). Let us assume $\gamma > \alpha$ and bound the first line of (29) in $\textsf {L}^{p}$. Using equation (31) to approximate $e^{-\gamma \mu _i}$, the fact that $\mathbbm {E}\left[ Y_i^{p\gamma }\right] =\frac{\textsf {B}(1+p\gamma -\alpha ,\theta +i\alpha )}{\textsf {B}(1-\alpha ,\theta +i\alpha )}$ together with (32), and also using Lemma 5.4 to upperbound $\left\Vert e^{\gamma S_i} \right\Vert _p$, we obtain that for some constant $C>0$ and any $p\ge 1$,

$$\begin{aligned} \left\Vert \zeta _{N,\gamma } \right\Vert _p&\le \sum _{i=1}^N \left\Vert Y_i^\gamma \right\Vert _p e^{-\gamma \mu _{i-1}}\left\Vert e^{\gamma S_{i-1}} \right\Vert _p\\&\le C \left\Vert e^{\gamma S_\infty } \right\Vert _p \sum _{i=1}^\infty i^{-\gamma } i^{\gamma -{\gamma }/{\alpha }} \left( 1+o\big (1\big )\right) <\infty . \end{aligned}$$

In particular the increasing sequence $\left( \zeta _{N,\gamma }^p\right) _{N\in \mathbb {N}}$ is a.s. bounded and thus convergent. The convergence also holds in $\textsf {L}^{p}$ by dominated convergence which yields $\left\Vert \zeta _{N,\gamma } \right\Vert _{{p}} \rightarrow \left\Vert \zeta _{\infty ,\gamma } \right\Vert _{{p}}$. $\square$

### Proof of Theorem 5.1

In this section we gather the results obtained so far in order to prove Theorem 5.1. Before we do so, we recall yet another well-known fact on the Poisson–Dirichlet distribution, mainly that removing the element ${\tilde{V}}_1$ from $({\tilde{V}}_1, {\tilde{V}}_2, \dots )$ and renormalizing the resulting sequence leads, in distribution, to a change of parameters.

#### Lemma 5.8

(Poisson–Dirichlet change of parameter) Let $({\tilde{V}}_1,{\tilde{V}}_2,\dots )$ be a size-biased pick from a PD$(\alpha ,\theta )$ partition. Then the sequence $\left( \frac{{\tilde{V}}_2}{1-{\tilde{V}}_1}, \frac{{\tilde{V}}_3}{1-{\tilde{V}}_1}, \dots \right)$ is distributed as a size-biased pick from a PD($\alpha , \theta + \alpha$) partition.

The martingale properties obtained in the previous section entail that $c_N$ can be compared to $\mathbbm {E}\left[ \left( \eta ^{(N)}_1\right) ^2\right]$.

#### Lemma 5.9

Assume $\alpha /2<\gamma \le \alpha$. Then there exists a constant $B\equiv B_{\alpha ,\theta ,\gamma }$ such that

$$\begin{aligned} c_N\ge Bu_N^{-2}. \end{aligned}$$

Moreover, when $$\theta \le 0$$

$$\begin{aligned} c_N\le \mathbbm {E}\left[ \left( \eta ^{(N)}_1\right) ^2\right] +o\big (u_N^{-\frac{\alpha +\theta }{\alpha }}\big ), \end{aligned}$$

whereas

$$\begin{aligned} c_N\le \mathbbm {E}\left[ \left( \eta ^{(N)}_1\right) ^2\right] + \mathcal {O}\left( u_N^{-2}\right) \end{aligned}$$

whenever $$\theta >0$$.

#### Proof

On the one hand, choose $p>3$. By the reverse Hölder’s inequality we have

$$\begin{aligned} c_N&= \mathbbm {E}_{\alpha ,\theta }\left[ \frac{\zeta _{N,2\gamma }}{\zeta _{N,\gamma }^2}\right] \ge \mathbbm {E}_{\alpha ,\theta }\left[ \frac{\zeta _{N,2\alpha }}{\zeta _{N,\gamma }^2}\right] \ge \mathbbm {E}_{\alpha ,\theta } \left[ {\zeta _{N,2\alpha }^\frac{1}{p}}\right] ^p\mathbbm {E}_{\alpha ,\theta }\left[ \zeta _{N,\gamma }^{\frac{2}{p-1}}\right] ^{1-p}, \end{aligned}$$

where $\mathbbm {E}_{\alpha ,\theta }$ refers to expectation with respect to the PD$(\alpha ,\theta )$ distribution. Thanks to Proposition 5.7 the first term in the r.h.s. above is of order $\mathcal {O}\left( 1\right)$ as $N\rightarrow \infty$; whereas the second term is of order $\mathcal {O}\left( u_N^{-\frac{2}{p-1}(1-p)}\right)$ (using $2\alpha >\alpha$ for the first, and $\frac{2\gamma }{(p-1)}>0>-(\alpha +\theta )$ for the second). We thus conclude that

$$\begin{aligned} c_N&\ge Bu_N^{-2} \end{aligned}$$

for some $$B>0$$.

On the other hand, by Lemma 5.8 in the second equality below, we have for any $\delta >0$,

37 $$\begin{aligned}&\mathbbm {E}_{\alpha ,\theta }\left[ \sum _{i=2}^N \left( \eta ^{(N)}_i\right) ^\delta \right] \nonumber \\ &= \mathbbm {E}_{\alpha ,\theta }\left[ \frac{\sum _{i=2}^N\left( \frac{{\tilde{V}}_{i}}{ 1-{\tilde{V}}_1}\right) ^{\delta \gamma }(1-{\tilde{V}}_1)^{\delta \gamma }}{ \left( {\tilde{V}}_1^\gamma + (1-{\tilde{V}}_1)^\gamma \sum _{j=2}^N \left( \frac{{\tilde{V}}_j}{1-{\tilde{V}}_1}\right) ^\gamma \right) ^\delta } \right] \nonumber \\&=\mathbbm {E}_{\alpha ,\theta +\alpha }\left[ \int _0^1 \frac{(1-y)^{\delta \gamma }\zeta _{N-1,2\gamma }}{\left( y^\gamma +(1-y)^\gamma \zeta _{N-1,\gamma }\right) ^\delta } \frac{y^{-\alpha }(1-y)^{\theta +\alpha -1}}{\textsf {B}(1-\alpha ,\theta +\alpha )} dy\right] \nonumber \\&=\mathbbm {E}_{\alpha ,\theta +\alpha }\left[ \frac{\zeta _{N-1,\delta \gamma }}{\zeta _{N-1,\gamma }^\delta } \int _0^1 \frac{(1-y)^{\delta \gamma }}{\left( y^\gamma /\zeta _{N-1,\gamma }+(1-y)^\gamma \right) ^\delta } \frac{y^{-\alpha }(1-y)^{\theta +\alpha -1}}{\textsf {B}(1-\alpha ,\theta +\alpha )} dy \right] \nonumber \\&\le \mathbbm {E}_{\alpha ,\theta +\alpha }\left[ \frac{\zeta _{N-1,\delta \gamma }}{\zeta _{N-1,\gamma }^\delta }\right] . \end{aligned}$$

If we set $\frac{\alpha }{\gamma }<\delta <\frac{2\alpha +\theta }{\gamma }$ and $p=1+\epsilon ,q=\frac{p}{p-1}$ with $\epsilon >0$ small enough to ensure $p\delta \gamma <2\alpha +\theta$, we can use Proposition 5.7. By (33), with $\eta =p\delta$, we get $\mathbbm {E}_{\alpha ,\theta +\alpha }\left[ \zeta _{N-1,\gamma }^{-p\delta }\right] ^{\frac{1}{p}}=\mathcal {O}\left( \left( u_N\right) ^{-\delta }\right)$. By (34), and observing that $\delta \gamma >\alpha$, we get $\mathbbm {E}_{\alpha ,\theta +\alpha }\left[ \zeta _{N-1,\delta \gamma }^q\right] ^{\frac{1}{q}}=\mathcal {O}\left( 1\right)$. Thus, since $\gamma >\alpha /2$ we may choose $\delta \in [0,2]\cap \left( \frac{\alpha }{\gamma },\frac{2\alpha +\theta }{\gamma }\right) \ne \emptyset$ and use Hölder’s inequality, with *p* and *q* as before, in the last line above to obtain

$$\begin{aligned} \mathbbm {E}_{\alpha ,\theta }\left[ \sum _{i=2}^N \left( \eta ^{(N)}_i\right) ^2\right] \le \mathbbm {E}_{\alpha ,\theta }\left[ \sum _{i=2}^N \left( \eta ^{(N)}_i\right) ^\delta \right] =\mathcal {O}\left( u_N^{-\delta }\right) . \end{aligned}$$

In particular, since $\gamma \le \alpha$, for $\theta \le 0$ we may conclude $\mathbbm {E}_{\alpha ,\theta }\left[ \sum _{i=2}^N \left( \eta ^{(N)}_i\right) ^2\right] =o\big (u_N^{-\frac{\alpha +\theta }{\alpha }}\big )$, whereas for $\theta >0$ we obtain $\mathbbm {E}_{\alpha ,\theta }\left[ \sum _{i=2}^N \left( \eta ^{(N)}_i\right) ^2\right] =\mathcal {O}\left( u_N^{-2}\right)$. $\square$

We now prove the stated convergence of the genealogy.

#### Proof of Theorem 5.1

We first derive asymptotics for $\mathbbm {E}_{\alpha ,\theta }\left[ \left( \eta ^{(N)}_1\right) ^\delta \right]$ where $\delta >0$. By Lemma 5.8 we have

$$\begin{aligned}&\mathbbm {E}_{\alpha ,\theta }\left[ \left( \eta ^{(N)}_1\right) ^\delta \right] \\&\quad =\mathbbm {E}_{\alpha ,\theta +\alpha } \left[ \int _0^1 \frac{y^{\gamma \delta }}{\left( y^\gamma +(1-y)^\gamma \zeta _{N-1,\gamma }\right) ^\delta } y^{-\alpha }(1-y)^{\theta +\alpha -1} \frac{dy}{\textsf {B}(1-\alpha ,\theta +\alpha )}\right] , \end{aligned}$$

where, making the change of variable $u=(1-y)^\gamma \zeta _{N-1,\gamma }$, we obtain

38 $$\begin{aligned}&\mathbbm {E}_{\alpha ,\theta }\left[ \left( \eta ^{(N)}_1\right) ^\delta \right] =\frac{1}{\gamma \textsf {B}(1-\alpha ,\theta +\alpha )}\times \nonumber \\&\quad \mathbbm {E}_{\alpha ,\theta +\alpha }\left[ \frac{ 1 }{\zeta _{N-1,\gamma }^{1+\frac{\theta }{\alpha }}} \int _0^{\zeta _{N-1,\gamma }} \frac{\left( 1-\frac{u^{\frac{1}{\gamma }}}{\zeta _{N-1,\gamma }^{\frac{1}{\gamma }}}\right) ^{\gamma \delta }}{\left( \left( 1-\frac{u^{\frac{1}{\gamma }}}{\zeta _{N-1,\gamma }^{\frac{1}{\gamma }}}\right) ^{\gamma }+ u\right) ^\delta } \left( 1-\frac{u^{\frac{1}{\gamma }}}{\zeta _{N-1,\gamma }^{\frac{1}{\gamma }}}\right) ^{-\alpha } u^{\frac{\theta }{\alpha }} du \right] . \end{aligned}$$

Denote by $I_N\equiv I_{N,\delta }$ the integral inside the expectation above. Choose $0<\epsilon <1$. Then $I_N$ can be bounded on the set $\left\{ \frac{u^{1/\gamma }}{\zeta _{N-1,\gamma }^{1/\gamma }}\le \epsilon \right\}$ by

$$\begin{aligned} \int _0^\infty \frac{u^{\frac{\theta }{\alpha }}}{((1-\epsilon )^\gamma + u)^\delta }du <\infty \end{aligned}$$

whenever $\delta >1+\frac{\theta }{\alpha }$. On the other hand, on the set $\left\{ \frac{u^{1/\gamma }}{\zeta _{N-1,\gamma }^{1/\gamma }}>\epsilon \right\}$ the integral $I_N$ can be bounded by

$$\begin{aligned} \int _{\epsilon ^\gamma \zeta _{N-1,\gamma }}^{\zeta _{N-1,\gamma }} (1-\epsilon )^{2\gamma - \alpha }u^{\frac{\theta }{\alpha }-\delta }du \le C \zeta _{N-1, \gamma }^{1+\frac{\theta }{\alpha }-\delta } \end{aligned}$$

for some $C>0$. These two bounds, together with the fact that $\zeta _{N-1,\gamma }\rightarrow \infty$ as $N\rightarrow \infty$ (from Proposition 5.7), allow us to use (pathwise) dominated convergence to obtain, for $\delta >1+\frac{\theta }{\alpha }$ and as $N\rightarrow \infty$,

$$\begin{aligned} I_N {\rightarrow } \int _0^\infty \frac{u^{\frac{\theta }{\alpha }}}{(1+u)^\delta }du \end{aligned}$$

almost surely. On the other hand, substituting these bounds into the expectation in (38) we obtain $\mathbbm {E}_{\alpha ,\theta }\left[ \left( \eta ^{(N)}_1\right) ^\delta \right] = \mathcal {O}\left( \mathbbm {E}_{\alpha ,\alpha +\theta }\left[ \zeta _{N-1,\gamma }^{1+\frac{\theta }{\alpha }}\right] \right) + \mathcal {O}\left( \mathbbm {E}_{\alpha ,\alpha +\theta }\left[ \zeta _{N-1,\gamma }^{-\delta }\right] \right)$ for $\delta >1+\frac{\theta }{\alpha }$. By (33) in Proposition 5.7 together with the fact that $\frac{\alpha +\theta }{\alpha }\gamma <2\alpha +\theta$, the first term is of order $\mathcal {O}\left( u_N^{-1-\frac{\theta }{\alpha }}\right)$. If furthermore $\delta <\frac{2\alpha +\theta }{\gamma }$ then, again by (33), the second term is of order $\mathcal {O}\left( u_N^{-\delta }\right)$. Since the random variable $\left( \eta ^{(N)}_1\right) ^\delta$ is decreasing in $\delta$, the condition $\delta <\frac{2\alpha +\theta }{\gamma }$ was no restriction, and we may conclude $\mathbbm {E}_{\alpha ,\theta }\left[ \left( \eta ^{(N)}_1\right) ^\delta \right] =\mathcal {O}\left( u_N^{-1-\frac{\theta }{\alpha }}\right)$ for all $\delta >1+\frac{\theta }{\alpha }$. Thus we may apply dominated convergence in (38) which, together with Proposition 5.7, yield for $\delta >1+\frac{\theta }{\alpha }$,

39 $$\begin{aligned}&\lim _{N\rightarrow \infty } u_N^{1+\frac{\theta }{\alpha }}\mathbbm {E}_{\alpha ,\theta }\left[ \left( \eta ^{(N)}_1\right) ^\delta \right] \\&\quad = \mathbbm {E}_{\alpha ,\alpha +\theta }\left[ \left( \frac{\Gamma (1+\gamma -\alpha )}{\Gamma (1-\alpha )}K_{\alpha ,\theta }^{-\gamma }e^{\gamma S_\infty }\right) ^{-1-\frac{\theta }{\alpha }} \right] \frac{\gamma ^{-1}}{\textsf {B}(1-\alpha ,\alpha +\theta )} \int _0^\infty \frac{u^{\frac{\theta }{\alpha }}}{(1+u)^\delta }du.\nonumber \end{aligned}$$

We now turn to the case (i). Given Lemma 5.9, and the condition $\theta \in (-\alpha ,\alpha )$, it remains to prove the condition (21) in Proposition 3.1. This is, in our context,

40 $$\begin{aligned} \lim _{N\rightarrow \infty } u_N^{1+\frac{\theta }{\alpha }}\mathbbm {E}_{\alpha ,\theta }\left[ \left( \eta ^{(N)}_1\right) ^b\right] = \ell _{\alpha ,\theta ,\gamma }\int _0^1 p^{b-2}\Lambda (dp) \end{aligned}$$

where $\Lambda (dp)$ is the coagulation measure of the Beta$(1+\frac{\theta }{\alpha }, 1-\frac{\theta }{\alpha })$-coalescent. When $\theta \in \left( -\alpha ,\alpha \right)$ we have $1+\frac{\theta }{\alpha }<2$. Thus (39) holds for all $\delta =b\ge 2.$ By means of the change of variable $x=(1+u)^{-1}$ we may rewrite the integral in (39) with $\delta =b$ as

$$\begin{aligned} \int _0^\infty \frac{u^{\frac{\theta }{\alpha }}}{(1+u)^b}du = \int _0^1 x^{b-2}x^{1-\frac{\theta }{\alpha }-1}(1-x)^{1+\frac{\theta }{\alpha }-1} dx. \end{aligned}$$

On the other hand, by Lemma 5.4,

$$\begin{aligned}&\mathbbm {E}_{\alpha ,\alpha +\theta }\left[ \left( \frac{\Gamma (1+\gamma -\alpha )}{\Gamma (1-\alpha )}K_{\alpha ,\theta }^{-\gamma }e^{\gamma S_\infty }\right) ^{-1-\frac{\theta }{\alpha }} \right] \frac{\gamma ^{-1}}{\textsf {B}(1-\alpha ,\alpha +\theta )}\\&\quad =\gamma ^{-1}\left( \frac{\Gamma (1-\alpha )}{\Gamma (1+\gamma -\alpha )}\right) ^{1+\frac{\theta }{\alpha }} \frac{\Gamma (\alpha +\theta +1)}{\Gamma \left( \alpha +\theta -\frac{\gamma }{\alpha }(\alpha +\theta ) +1\right) } \nonumber \\&\qquad \times \frac{\Gamma \left( \frac{1}{\alpha }(\alpha +\theta -\frac{\gamma }{\alpha }(\alpha +\theta ))+1\right) }{\Gamma \left( \frac{\alpha +\theta }{\alpha }+1\right) } \frac{\Gamma (1+\theta )}{\Gamma (1-\alpha )\Gamma (\alpha +\theta )}\\&\quad = \frac{\alpha }{\gamma }\frac{\Gamma (1-\alpha )^{\frac{\theta }{\alpha }}}{\Gamma (1+\gamma -\alpha )^{1+\frac{\theta }{\alpha }}} \frac{\Gamma \left( \frac{\alpha +\theta }{\alpha }(1-\frac{\gamma }{\alpha })+1\right) }{\Gamma \left( \left( \alpha +\theta \right) \left( 1-\frac{\gamma }{\alpha }\right) +1\right) } \frac{\Gamma (1+\theta ) \Gamma \left( 1-\frac{\theta }{\alpha }\right) }{\textsf {B}\left( 1-\frac{\theta }{\alpha },1+\frac{\theta }{\alpha }\right) }. \end{aligned}$$

Substituting both in (39) we obtain (40).

Let us now turn to case (ii); we will prove the conditions of Proposition 3.2. For the first condition, recall that, by Lemma 5.9, $c_N\ge Cu_N^{-2}$ for some $C>0$. Then, since $\frac{\alpha }{\gamma }<2$ and $\frac{2\alpha +\theta }{\gamma }\ge 3$; the argument after equation (37) yields for $2<\delta <3$ (and any $\theta \ge \alpha$),

$$\begin{aligned} \mathbbm {E}_{\alpha ,\theta }\left[ \sum _{i=2}^N \left( \eta ^{(N)}_i\right) ^3\right] =\mathcal {O}\left( u_N^{-\delta }\right) =o\big (c_N\big ). \end{aligned}$$

It remains to prove the second condition of Proposition 3.2. When $\theta >\alpha$ we also have, choosing $\delta >1+\frac{\theta }{\alpha }\ge 2$ in (39), that $\mathbbm {E}_{\alpha ,\theta }[\left( \eta ^{(N)}_1\right) ^\delta ]=\mathcal {O}\left( u_N^{-1-\frac{\theta }{\alpha }}\right) =o\big (c_N\big )$. The convergence of the genealogy then follows in this case.

The special case when $\theta =\alpha$ needs to be treated separately. Observe that for $\delta = 1+\frac{\theta }{\alpha }=2$ we have the following bounds on $I_N$ (for some $C_1,C_2\in \mathbb {R}$)

$$\begin{aligned} C_1+\int _2^{\zeta _{N-1,\gamma }}\frac{\left( 1-\frac{u}{\zeta _{N-1,\gamma }}\right) ^{2}}{(\frac{1}{u}+1)^2}u^{-1}du \le I_N \le C_2+\int _2^{\zeta _{N-1,\gamma }}u^{-1}du. \end{aligned}$$

These, together with the fact that (by an application of the Mean Value Theorem) $\left|1-\left( 1-\frac{u}{\zeta _{N-1,\gamma }}\right) ^{2} \right| \le u/\zeta _{N-1,\gamma }$, and that $\lim _{x\rightarrow \infty }\frac{1}{\log (x)}\int _2^x\frac{1}{(1/u+1)^2}u^{-1}du=\lim _{x\rightarrow \infty }\frac{1}{\log (x)}\int _2^xu^{-1}du$, yield using dominated convergence,

$$\begin{aligned} \lim _{N\rightarrow \infty }\frac{I_N}{\log (\zeta _{N-1,\gamma }\vee 2)}{=} \lim _{N\rightarrow \infty } \frac{1}{\log (\zeta _{N-1,\gamma }\vee 2)}\int _2^{\zeta _{N-1,\gamma }}u^{-1}du =1 \end{aligned}$$

almost surely. Since $I_N\le C_2+\log (\zeta _{N-1,\gamma }\vee 2)$, the above convergence also holds in $\textsf {L}^{p}$,$p>0$. On the other hand, by Proposition 5.7, the limit $\lim _{N\rightarrow \infty }\log \left( (\zeta _{N-1,\gamma }\vee 2) / u_N^{1+\frac{\theta }{\alpha }}\right)$ exists almost surely and in $\textsf {L}^{p},p>0$. Thus

$$\begin{aligned} \lim _{N\rightarrow \infty }\frac{\log \left( \zeta _{N-1,\gamma }\vee 2\right) }{(1+\frac{\theta }{\alpha })\log \left( u_N\right) } =1. \end{aligned}$$

This implies, multiplying and dividing the l.h.s. below by $\log (\zeta _{N-1,\gamma }\vee 2)$,

$$\begin{aligned}\lim _{N\rightarrow \infty }\frac{I_N}{(1+\frac{\theta }{\alpha })\log \left( u_N\right) }&=\lim _{N\rightarrow \infty }\log \left( (\zeta _{N-1,\gamma }\vee 2)/u_N^{1+\frac{\theta }{\alpha }}\right) \\&=\log \left( \frac{\Gamma (1+\gamma -\alpha )}{\Gamma (1-\alpha )}K^{-\gamma }_{\alpha ,\theta }e^{\gamma S_\infty }\right) \end{aligned}$$

almost surely and in $\textsf {L}^{p}$, $p>0$. Plugging into (38) we then obtain, choosing *p* close enough to 1 so that $p(1+\frac{\theta }{\alpha })=2p<\frac{2\alpha +\theta }{\gamma }$ and $q=\frac{p}{p-1}$,

$$\begin{aligned} \mathbbm {E}_{\alpha ,\theta }\left[ \left( \eta ^{(N)}_1\right) ^2\right]&\le \mathbbm {E}_{\alpha ,\alpha +\theta }\left[ \zeta _{N-1,\gamma }^{-2p}\right] ^{1/p} \mathbbm {E}_{\alpha ,\alpha +\theta }\left[ I_N^q\right] ^{1/q}\\&=\mathcal {O}\left( u_N^{-2}\log \left( u_N\right) \right) . \end{aligned}$$

By dominated convergence in (38), the latter implies

$$\begin{aligned} \lim _{N\rightarrow \infty }\frac{u_N^{2}}{\log \left( u_N\right) } \frac{\mathbbm {E}_{\alpha ,\alpha +\theta }\left[ \left( \eta ^{(N)}_1\right) ^2\right] }{1+\frac{\theta }{\alpha }} =\mathbbm {E}\left[ \frac{\log \left( \frac{\Gamma (1+\gamma -\alpha )}{\Gamma (1-\alpha )}K_{\alpha ,\theta }^{-\gamma }e^{\gamma S_\infty }\right) }{\left( \frac{\Gamma (1+\gamma -\alpha )}{\Gamma (1-\alpha )}K_{\alpha ,\theta }^{-\gamma }e^{\gamma S_\infty }\right) ^{2}}\right] . \end{aligned}$$

By Lemma 5.9 we thus have $u_N^{-2}=o\big (\mathbbm {E}_{\alpha ,\theta }\left[ \left( \eta ^{(N)}_1\right) ^2\right] \big )=o\big (c_N\big )$. Then, choosing $\delta >1+\frac{\theta }{\alpha }$ in (39) we obtain $\mathbbm {E}\left[ \left( \eta ^{(N)}_1\right) ^\delta \right] =o\big (c_N\big )$. The proof is completed by a second application of Proposition 3.2. $\square$

## Data Availability

Data sharing not applicable to this article as no datasets were generated or analysed during the current study.
